# *RNF128* deficiency in macrophages promotes colonic inflammation by suppressing the autophagic degradation of S100A8

**DOI:** 10.1038/s41419-025-07338-0

**Published:** 2025-01-15

**Authors:** Xianwen Ran, Yue Li, Yahui Ren, Weilong Chang, Rui Deng, Huifen Wang, Weiwei Zhu, Yize Zhang, Yudie Cai, Jia Hu, Di Wang, Zhibo Liu

**Affiliations:** 1 Department of Gastrointestinal Surgery, The First Affiliated Hospital of Zhengzhou University, Zhengzhou, 450052 China; 2 Translational Medicine Center, The First Affiliated Hospital of Zhengzhou University, Zhengzhou, 450052 China; 3 Department of Cardiology, The First Affiliated Hospital of Zhengzhou University, Zhengzhou, Henan 450052 China; 4 Department of Pediatrics, Union Hospital, Tongji Medical College, Huazhong University of Science and Technology, Wuhan, 430022 China; 5 Department of Infectious Diseases, The First Affiliated Hospital of Zhengzhou University, Zhengzhou, 450052 China; 6 Gene Hospital of Henan Province, The First Affiliated Hospital of Zhengzhou University, Zhengzhou, 450052 China; 7 Institute of Biology and Medicine, College of Life and Health Sciences, Wuhan University of Science and Technology, Wuhan, 430081 China

**Keywords:** Inflammation, Proteins

## Abstract

Macrophages play important roles in maintaining intestinal homeostasis and in the pathogenesis of inflammatory bowel diseases (IBDs). However, the underlying mechanisms that govern macrophage-mediated inflammation are still largely unknown. In this study, we report that RNF128 is downregulated in proinflammatory macrophages. *RNF128* deficiency leads to elevated levels of effector cytokines in vitro and accelerates the progression of IBD in mouse models. Bone marrow transplantation experiments revealed that *RNF128* deficiency in bone marrow cells contributes to the worsening of DSS-induced colitis. Mechanistically, RNF128 interacts with and destabilizes S100A8 by promoting its autophagic degradation, which is mediated by the cargo receptor Tollip. Moreover, the administration of an S100A8 neutralizing antibody mitigated the development of colitis and improved survival in DSS-treated *Rnf128*^*−/−*^ mice. Overall, our study underscores the anti-inflammatory role of RNF128 in macrophages during the progression of colitis and highlights the potential of targeting the RNF128-Tollip-S100A8 axis to attenuate intestinal inflammation for the treatment of colitis.

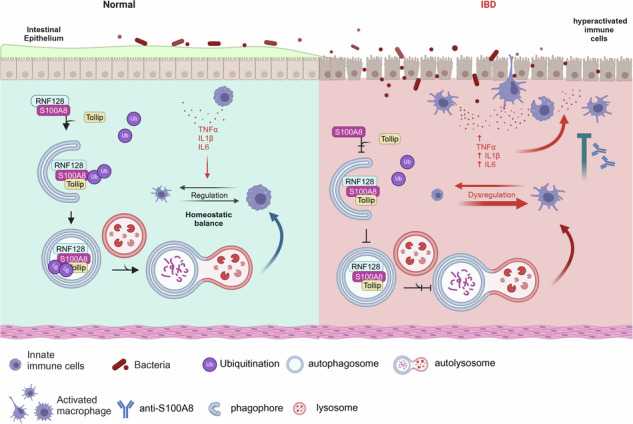

## Introduction

Inflammatory bowel diseases (IBDs), including Crohn’s disease (CD) and ulcerative colitis (UC), are characterized by relapsing inflammation and dysregulated immune responses in the digestive tract [1, 2]. IBD patients usually have poor physical and mental health and are at increased risk of developing colorectal cancer (CRC) [3]. Intestinal resident macrophages play crucial roles in fine-tuning the mucosal immune system by clearing pathogens, toxins, and dead cells [4, 5]. However, excessive macrophage infiltration and activation in the intestine lead to the erosion of intestinal epithelial cells and the disruption of intestinal homeostasis during IBD [6]. Thus, targeting intestinal macrophages represents a promising therapeutic approach for controlling intestinal inflammation and restoring tissue function [7, 8]. It is of great value to elucidate the mechanisms that govern macrophages-mediated inflammation in IBD and identify new therapeutic targets.

RNF128, also known as gene related to energy in lymphocytes (GRAIL), is a member of the RING domain-containing E3 ligases and has been shown to have important functions in both innate immunity and adaptive T cell responses, thus influencing various physiological or pathological processes, such as inflammation, tumor and infection [9–12]. Several studies suggest that there is a link between RNF128 and IBDs. RNF128 was downregulated in peripheral blood CD4 + T cells while upregulated in the intestinal lamina propria in CD patients. Intravenous injection of RNF128-overexpressing T cells significantly alleviated the severity of colitis in a mouse model [13]. In UC patients, RNF128 expression was elevated during remission and associated with clinical improvement following treatment [14]. A recent study reported that *RNF128* deficiency in intestinal epithelial cells promoted colitis and colorectal tumorigenesis by activating IL-6-STAT3 signaling [15]. However, the roles as well as the mechanism of macrophage RNF128 in IBD are still unexplored.

In this study, we found that RNF128 was downregulated in proinflammatory macrophages. The decreased expression of RNF128 in macrophages was responsible for the exacerbation of IBD. Mechanistically, RNF128 facilitated the K63-linked ubiquitination of S100A8 at its lysine 36, which is recognized by the cargo receptor Tollip and leads to selective autophagic degradation of S100A8. Neutralizing antibodies against S100A8 significantly alleviated the exacerbation of colitis in DSS-treated *Rnf128*^*−/−*^ mice. In summary, our study reveals the functional roles and mechanisms of macrophage RNF128 in IBD progression, which may provide novel targets and approaches for IBD treatment.

## Materials and methods

### Antibodies and reagents

The antibodies and reagents used in this study are listed in Table S1.

### Cell culture and transfection

HEK293T and THP-1 cells were purchased from American Type Culture Collection (ATCC). HEK293T cells were cultured in DMEM supplemented with 10% FBS, and THP-1 cells in RPMI-1640 supplemented with 10% FBS. THP-1 cells were treated with 100 ng/ml PMA for 24 h to differentiate into macrophages.

Bone marrow-derived macrophages (BMDMs) from *Rnf128*^*+/+*^ and *Rnf128*^*−/−*^ mice were obtained according to previously described [16]. Briefly, the femurs and tibias of the mice were flushed with DMEM containing penicillin (100 units/mL) and streptomycin (100 μg/mL). Bone marrow cells were collected and cultured in complete DMEM (with 10% FBS) supplemented with 10 ng/ml M-CSF for 7 days.

All cells mentioned above were incubated at 37 °C in an incubator with 5% CO_2_. The cells were transfected with plasmids or siRNA via Lipofectamine 3000 following the instruction manual. The siRNA sequences used in our studies are shown in Table S2.

### Human samples

Colitis biopsy samples and colitis blood samples were collected from UC patients from the First Affiliated Hospital of Zhengzhou University. Normal adjacent colon tissues were collected from colon cancer patients admitted for colectomy surgery. Normal blood samples were obtained from healthy volunteers. This study was approved by the Human Ethical Committee of the First Affiliated Hospital of Zhengzhou University (*2023-KY-0423*).

### Plasmid construction

Human RNF128 or S100A8 cDNA was amplified from THP-1 cells and cloned into indicated vectors. RNF128 or S100A8 truncations were constructed based on wild-type plasmid using polymerase chain reaction (PCR). HA-Ub (WT, K6, K11, K27, K29, K33, K48, and K63) strains were obtained from Addgene. All plasmids in our study were confirmed by DNA sequencing. The primer sequences for the plasmid construction are shown in Table S3.

### Lentiviral transduction

HEK293T cells were co-transfected with the indicated lentiviral vectors, psPAX2 and pMD2. G plasmids to produce lentiviral particles. THP-1 cells were infected with lentiviral particles and selected with puromycin (1 μg/mL). The knockdown or knockout efficiency were identified by genome DNA sequencing and western blot. The shRNA and sgRNA sequences used in the study are listed in Table S4.

### Western blotting

Colon tissues or cells were lysed with RIPA buffer containing protease inhibitors. Protein concentrations were measured via a BCA kit. Approximately 30 μg of protein was electrophoresed on SDS–PAGE gels and transferred onto nitrocellulose membranes. The membranes were blocked with 5% skim milk and then incubated with primary antibodies overnight. After incubated with secondary antibodies, specific protein bands were visualized with an Odyssey Chemiluminescent Imaging System.

### Immunoprecipitation and co-immunoprecipitation

The cells were lysed by RIPA buffer containing protease inhibitors. The extract was incubated with indicated antibody and protein A/G Agarose beads at 4 °C. After being washed 3 times, the beads were boiled in protein loading buffer and subjected to western blot or liquid chromatography‒mass spectrometry (LC-MS/MS) analysis.

### Enzyme-linked immunosorbent assay

TNF-α and IL-1β levels in serum or colitis tissues of *Rnf128*^*+/+*^ or *Rnf128*^*−/−*^ mice were determined using a TNF-α ELISA Kit and an IL-1β ELISA Kit according to the manufacturer’s protocol.

### Immunofluorescence

For colitis tissue immunofluorescence staining, dewaxed and rehydrated tissue sections were subjected to antigen retrieval in citrate buffer. Endogenous peroxidases and non-specific antigens were blocked with 0.3% hydrogen peroxidase and 5% fetal bovine serum (FBS). For cell immunofluorescence staining, cells seeded in confocal plates were fixed with 4% paraformaldehyde, permeabilized with 0.2% Triton X-100 and blocked with 5% FBS. The blocked cells and tissue sections were incubated with indicated primary antibodies and fluorescently labeled secondary antibody. The nuclei were stained with Hoechst 33342. The cells and tissue sections were visualized with fluorescence or confocal microscopy (Zeiss).

### Transmission electron microscopy

Transmission electron microscopy was used to observe the number and shape of autophagosomes. Briefly, the cells were centrifuged at 3000 rpm, and the supernatant was discarded. The cell pellet was fixed by cacodylate-buffered 1% osmium tetroxide, dehydrated in ethanol series, embedded in Poly/Bed812 and sectioned to 60–80 nm. The sections were stained with uranyl acetate and lead citrate and then observed with an electron microscope (FEI Tecnai G20, USA).

### Animal studies

The mice used in our studies were housed under specific pathogen-free conditions. The animal studies were approved by the ethics committee of the First Affiliated Hospital of Zhengzhou University (*ZZU-ZZU-LAC20240628[04]*). *Rnf128*^+/−^ mice were generated via CRISPR/Cas9 technology with deletion of Rnf128 exon 2 (Nanjing University Model Animal Research Center). *Rnf128*^−/−^ mice were obtained from the copulation of *Rnf128*^+/−^ mice. The primers used for identifying mice genotyping are listed in Table S5.

For the DSS-induced colitis model, 8-week-old male *Rnf128*^*+/+*^ and *Rnf128*^*−/−*^ mice were administered with 2.5% DSS drinking water for 7 days. Colitis disease activity index (DAI) was calculated daily based on the following criteria: weight loss from baseline (score: 0, no weight loss; 1, 1–3% weight loss; 2, 3–6% weight loss; 3, 6–9% weight loss; 4, >9% weight loss); stool consistency (score: 0, normal; 1, soft but still formed; 2, soft and loose stools; 3, very soft and wet; 4, watery diarrhea); and blood (score: 0, normal; 1 and 2, focal bloody stool; 3, blood traces in stool; and 4, gross rectal bleeding).

For the bone marrow chimeric mouse model, recipient mice were subjected to irradiation at a dose of 6 Gy. A total of 5.0 × 10^6^ donor bone marrow cells were injected into each recipient mouse via tail vein (i.v.). After 7 weeks, the chimeric mice were treated with DSS to induce colitis. The effect of transplantation was detected by western blot.

For the macrophage depletion model, 200 µL of clodronate liposomes were injected intravenously into mice on Days 1, 3, and 5 to deplete intestinal macrophages according to the manufacturer’s recommendations. The mice were treated with 2.5% DSS the day after the last injection for 7 days.

### Neutralizing antibody treatment

The neutralizing antibody against S100A8 was produced via the classical hybridoma‒monoclonal antibody technique (Beijing BGI Protein Research Center). In brief, the full-length S100A8 protein was expressed via an Insect-Baculovirus expression system. The mice were intraperitoneally (i.p.) injected with 200 μg of S100A8 antibody on Days 0, 2, 4, and 6 after the initiation of 2.5% DSS treatment.

### Immunohistochemistry

For tissue immunohistochemistry, dewaxed and rehydrated tissue sections were subjected to antigen retrieval in citrate buffer. Endogenous peroxidases and non-specific antigens were blocked with 0.3% hydrogen peroxidase and 5% fetal bovine serum. The sections were subsequently incubated with the indicated primary antibodies. HRP-linked secondary antibodies and DAB were used for color development. ImageJ was used for quantification of immunohistochemistry-positive areas.

### Statistical analysis

Statistical analyses were performed with GraphPad Prism 9. Student’s t test was used to compare two groups of data. One-way ANOVA was used to compare more than two groups of data. Survival curves were generated via the Kaplan‒Meier method and compared via the log-rank test. The results are presented as the means ± SDs. *P* < 0.05 was considered significant.

## Results

### RNF128 is downregulated in proinflammatory macrophages

To identify pivotal genes involved in the pathogenesis and progression of IBD, we analyzed the differentially expressed genes from GSE4183 and found that RNF128 was significantly downregulated in the colonic mucosa of IBD patients compared with healthy controls (Figure S1A, B). This finding was confirmed by 6 other publicly available colitis datasets (GSE92415, GSE38713, GSE51785, GSE59071, GSE73661 and GSE87466) (Figure S1C). We also established an acute colitis mice model and observed that Rnf128 expression was markedly lower in colitis tissues than that in normal tissues (Figure S1D, E). These results suggest that decreased expression of RNF128 may be associated with IBD.

**Fig. 1:**
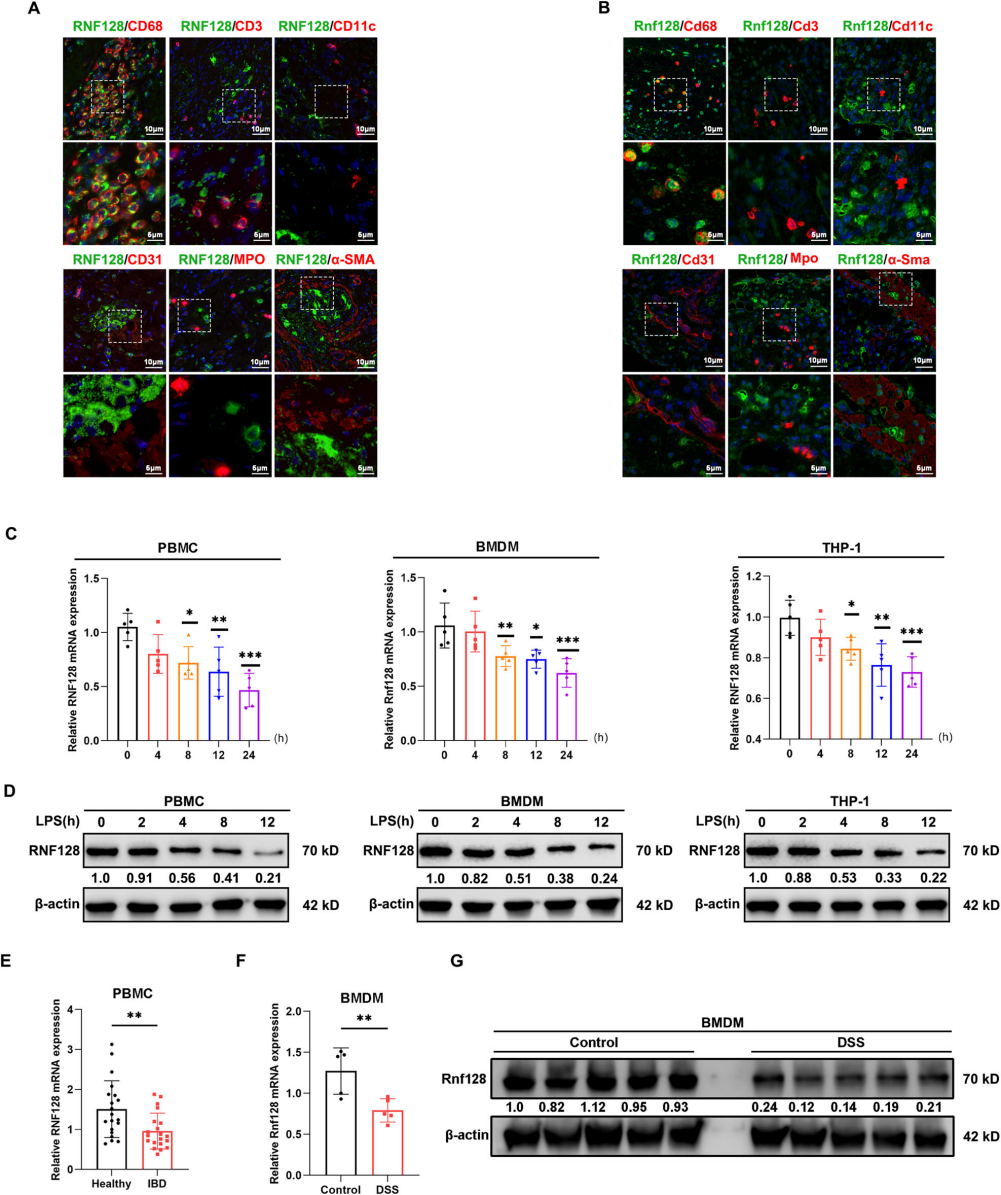
Inflammatory stimulation decreased RNF128 expression in macrophages. **A** Representative images of immunofluorescence co-staining for RNF128 (green) and the indicated cell-type markers (red) in human colitis tissues. DAPI (blue) was used to label the nuclei. Scale bars, 10 μm. **B** Representative images of immunofluorescence co-staining for Rnf128 (green) and the indicated cell-type markers (red) in colitis tissues from DSS-induced colitis mice. DAPI (blue) labels the nuclei. Scale bars, 10 μm. **C** Relative *RNF128* mRNA levels (normalized to GAPDH RNA levels) in PBMCs, BMDMs and THP-1 cells stimulated with LPS for different periods of time. Statistical data are presented as mean ± SD. *P < 0.05, **P < 0.01, ***P < 0.001. **D** Western blot analysis of RNF128 protein levels in PBMCs, BMDMs and THP-1 cells after LPS stimulation for different periods of time. **E** Relative *RNF128* mRNA levels (normalized to GAPDH RNA levels) in peripheral blood monocytes from healthy (*n* = 20) and patients with colitis (*n* = 20). Statistical data are presented as mean ± SD. **P < 0.01. **F** Relative *Rnf128* mRNA levels (normalized to GAPDH RNA levels) in BMDMs from control (*n* = 5) and DSS-induced colitis mice (*n* = 5). Statistical data are presented as mean ± SD. **P < 0.01. **G** Western blot analysis of Rnf128 protein levels in BMDMs from control (*n* = 5) and DSS-induced colitis mice (*n* = 5).

While previous studies have reported the function of RNF128 in colonic epithelial cells in IBD [15], its cellular sources and roles in other cell types remain largely unclear. We detected the location of RNF128 in different cell types within human and mice colitis tissues and found that RNF128 colocalized with CD68 and F4/80 (macrophage markers) but not with CD3 (lymphocyte marker), CD11c (dendritic cell marker), CD31 (endothelial marker), myeloperoxidase (MPO, neutrophil marker) or α-smooth muscle actin (α-SMA, fibroblast marker), indicating that RNF128 is expressed primarily in macrophages (Fig. 1A, B, Figure S1F). We further stimulated human peripheral blood mononuclear cells (PBMCs), mouse BMDMs and THP-1 cells with LPS and found that both the mRNA and protein levels of RNF128 were downregulated in a time-dependent manner (Fig. 1C, D). Moreover, RNF128 expression was lower in the PBMCs of IBD patients and the BMDMs of colitis mice than in those of healthy individuals or control mice (Fig. 1E–G). These results suggest that RNF128 expression is decreased in inflammatory macrophages.

### *RNF128* deficiency exacerbates intestinal inflammation in colitis model

**Fig. 2:**
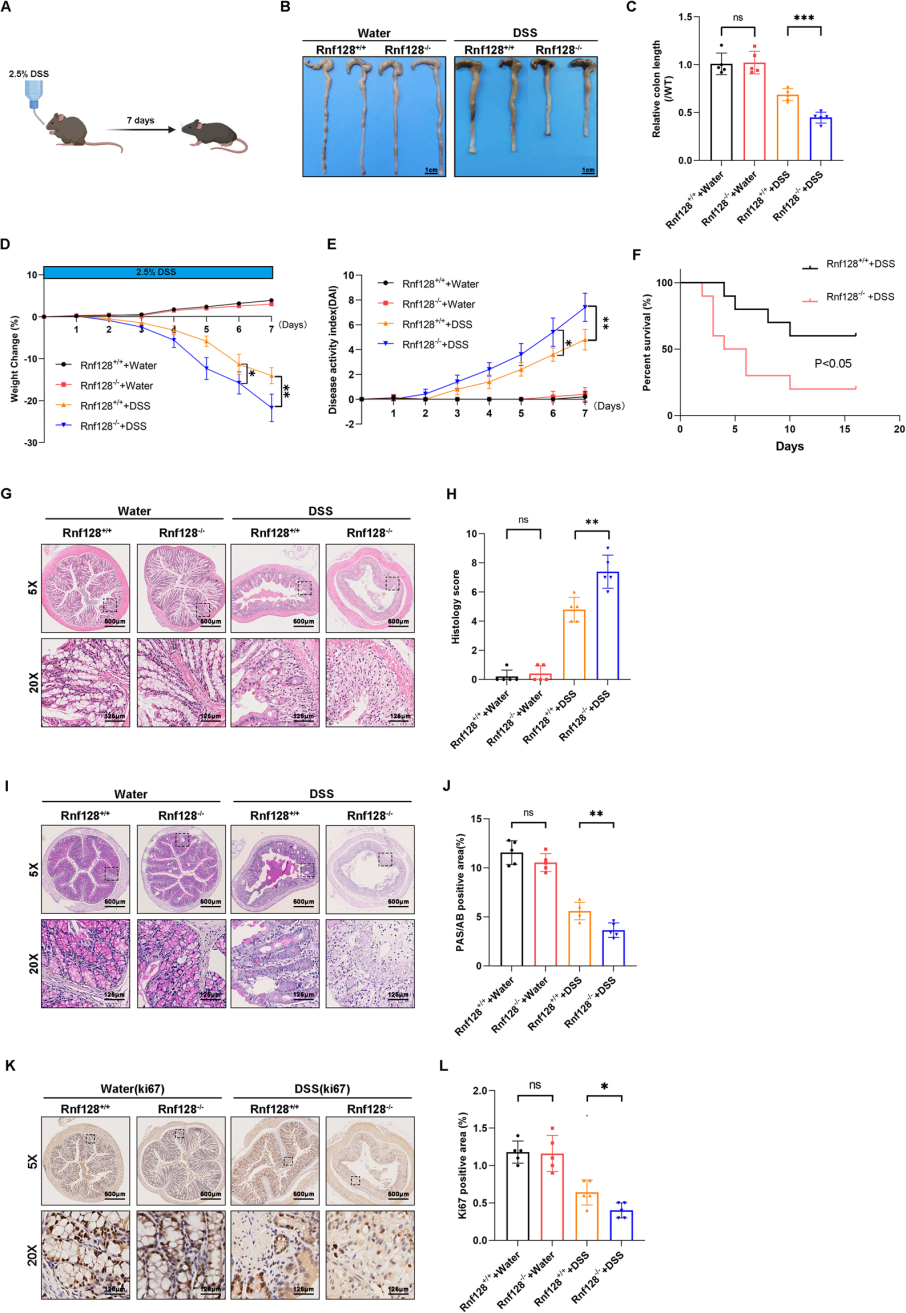
*Rnf128* deficiency aggravated DSS-induced colon injury. **A** Experimental scheme of DSS-induced colitis model. The mice were treated with 2.5% DSS drinking water for 7 consecutive days. **B** Representative images of colons from *Rnf128*^*+/+*^ (*n* = 5) and *Rnf128*^*−/−*^ (*n* = 5) mice after DSS treatment for 7 days. Scale bars, 1 cm. **C** The colon length in (**B**) was measured. Statistical data are presented as mean ± SD. ns nonsense, ***P < 0.001. **D** The body weight of the mice in (**B**) was measured daily. Statistical data are presented as mean ± SD. *P < 0.05, **P < 0.01. **E** Disease activity index scores of the mice in (**B**). Statistical data are presented as mean ± SD. *P < 0.05, **P < 0.01. **F** Survival analysis of *Rnf128*^*+/+*^ and *Rnf128*^*−/−*^ mice treated with water or 3% DSS (*n* = 10). Statistical data are presented as mean ± SD. *P < 0.05. **G** Representative H&E-staining images of colon sections from the mice in (**B**). Scale bar, 500 μm. **H** Histopathology score in (**G**) was measured. Statistical data are presented as mean ± SD. ns, nonsense, **P < 0.01. **I** Representative periodic acid sthiff-alcian blue (PAS/AB) staining images of colon sections from mice in (**B**). Scale bars, 500 μm. **J** The PAS-positive areas in (**I**) were measured and analyzed. Statistical data are presented as mean ± SD. ns, nonsense; **P < 0.01. **K** Representative Ki67 staining images of colon sections from mice in (**B**). Scale bars, 500 μm. **L** The Ki67 positive areas in (**K**) were measured and analyzed. Statistical data are presented as mean ± SD. ns nonsense. *P < 0.05.

To determine the role of RNF128 in colitis, we generated *Rnf128* knockout (*Rnf128*^*−/−*^) mice and established an acute colitis model by challenging *Rnf128*^*+/+*^ and *Rnf128*^*−/−*^ mice with 2.5% DSS (Fig. 2A, Figure S2A–C). Notably, *Rnf128* knockout did not affect intestinal development, and histological examination revealed no differences between *Rnf128*^*+/+*^ and *Rnf128*^*−/−*^ mice. However, *Rnf128*^*−/−*^ mice exhibited more severe intestinal inflammation in the DSS-induced colitis model, as evidenced by shorter colon length, increased weight loss and higher disease activity index (Fig. 2B–E). The survival rate of *Rnf128*^*−/−*^ mice was also significantly lower than that of *Rnf128*^*+/+*^ littermates with 3% DSS treatment (Fig. 2F). Accordingly, histopathologic staining revealed greater damage to the colonic epithelial mucosa and more extensive ulcerations in the DSS-treated *Rnf128*^*−/−*^ mice than in the control mice (Fig. 2G–J). There were fewer Ki67-positive cells in the intestinal crypts of Rnf128^−/−^ mice compared with those from *Rnf128*^*+/+*^ mice after DSS administration (Fig. 2K, L). We also established another chemically induced form of colitis using 2,4,6-trinitrobenzene sulfonic acid (TNBS) and observed similar intestinal inflammatory phenotypes in *Rnf128*^*−/−*^ mice (Figure S2D–I). These data demonstrate that knockout of *Rnf128* accelerates the progression of colitis.

### *Rnf128* deficiency enhances the production of proinflammatory cytokines in colitis model

**Fig. 3:**
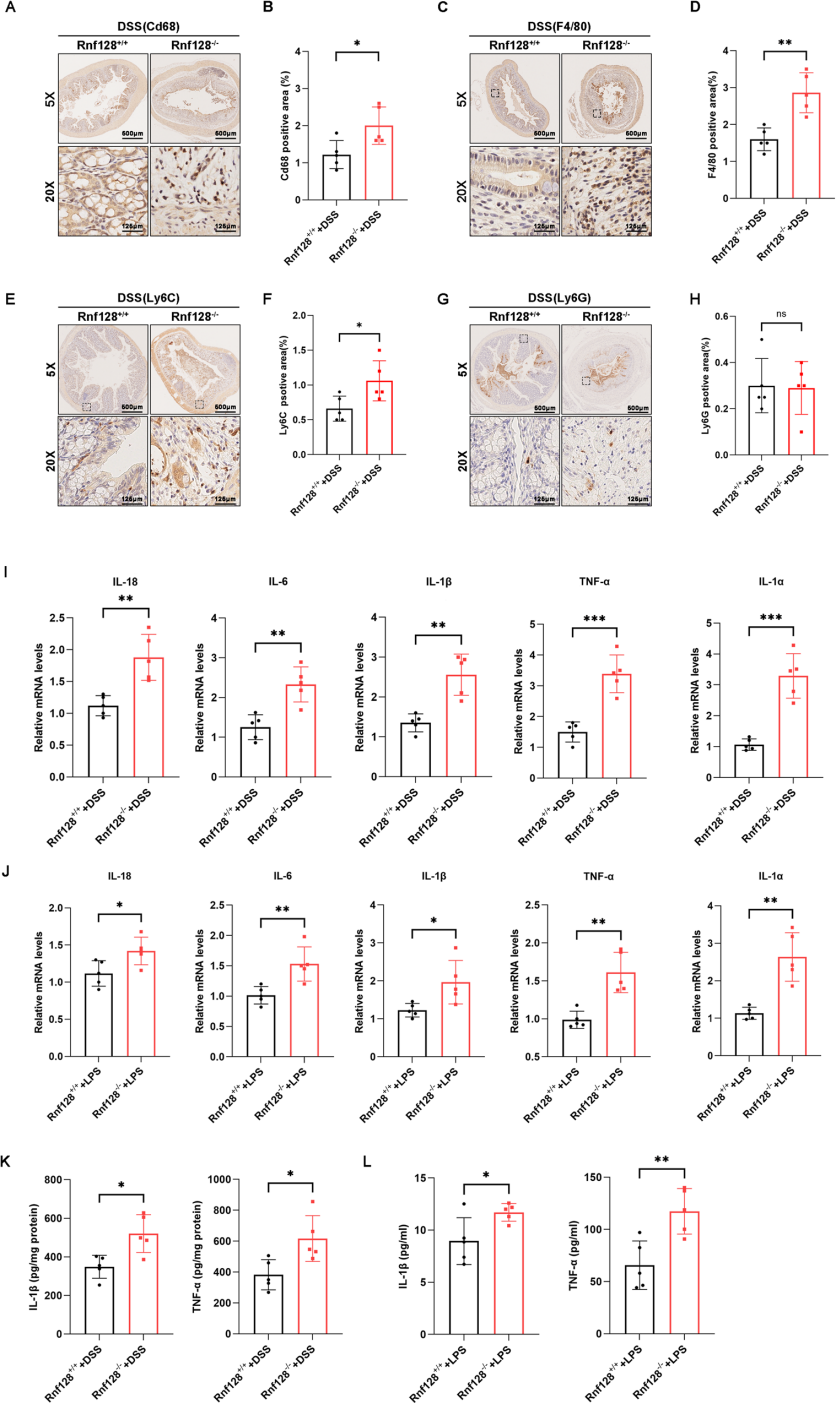
*RNF128* deficiency augments macrophage infiltration and inflammatory factors production. **A** *Rnf128*^*+/+*^ (*n* = 5) and *Rnf128*^*−/−*^ (*n* = 5) mice were exposed to 2.5% DSS for 7 days. Representative histochemical images of Cd68 staining in distal colon sections. Scale bars, 500 µm. **B** The staining positive areas in (**A**) were measured and analyzed. Statistical data are presented as mean ± SD. *P < 0.05. **C** *Rnf128*^*+/+*^ (*n* = 5) and *Rnf128*^*−/*−^ (*n* = 5) mice were exposed to 2.5% DSS for 7 days. Representative histochemical images of F4/80 staining in distal colon sections. Scale bar, 500 µm. **D** The F4/80 positive areas in (**C**) were measured and analyzed. Statistical data are presented as mean ± SD. **P < 0.01. **E** *Rnf128*^*+/+*^ (*n* = 5) and *Rnf128*^*−/*−^ (*n* = 5) mice were exposed to 2.5% DSS for 7 days. Representative histochemical images of Ly6C high staining in distal colon sections. Scale bars, 500 µm. **F** The Ly6C positive areas in (**E**) were measured and analyzed. Statistical data are presented as mean ± SD. *P < 0.05. **G** *Rnf128*^*+/+*^ (*n* = 5) and *Rnf128*^*−/−*^ (*n* = 5) mice were exposed to 2.5% DSS for 7 days. Representative histochemical images of Ly6G high staining in distal colon sections. Scale bars, 500 µm. **H** The Ly6G positive areas in (**G**) were measured and analyzed. Statistical data are presented as mean ± SD. ns nonsense. **I** The mRNA levels of Il18, Il6, Il1β, Il1α and Tnf-α (each normalized to GAPDH RNA level) in colon tissues from *Rnf128*^*+/+*^ (*n* = 5) and *Rnf128*^*–/–*^ (*n* = 5) mice subjected to 2.5% DSS. Statistical data are presented as mean ± SD. *P < 0.05, **P < 0.01, ***P < 0.001. **J** BMDMs were extracted from *Rnf128*^*+/+*^ and *Rnf128*^*–/–*^ mice and stimulated with LPS (200 ng/ml) for 8 h. The mRNA levels of Il18, Il6, Il1β, Il1α and Tnf-α (each normalized to GAPDH RNA level) in the BMDMs were detected via qRT‒PCR. The statistical data are presented as mean ± SD. *P < 0.05, **P < 0.01. **K** ELISA analysis of IL-1β and TNF-α levels in colon tissue from *Rnf128*^*+/+*^ (*n* = 5) and *Rnf128*^*–/–*^ (*n* = 5) mice subjected to 2.5% DSS. Statistical data are presented as mean ± SD. *P *<* 0.05. **L** ELISA analysis of IL-1β and TNF-α levels in the supernatant of BMDMs. Statistical data are presented as mean ± SD. *P < 0.05, **P < 0.01.

The pathological process of IBD is characterized by chronic relapsing intestinal inflammation [17]. *Rnf128* deficiency substantially increased macrophage infiltration in colonic tissues during colitis (Fig. 3A–D). Notably, Ly6C-high macrophage infiltration was increased in the intestines of *Rnf128*^*−/−*^ mice, but the number of Ly6G-positive cells did not significantly differ between the two groups (Fig. 3E–H). Compared with those in the control group, the mRNA levels of proinflammatory cytokines, such as Il18, Il1α, Il1β, Il6, and Tnf-α, were dramatically elevated in the colonic tissues of the DSS-treated *Rnf128*^−*/−*^ mice (Fig. 3I). Additionally, BMDMs from *Rnf128*^*−/−*^ mice exhibited higher levels of Il18, Il1α, Il1β, Il6 and Tnf-α compared to wild-type BMDMs upon LPS stimulation (Fig. 3J). The protein levels of Il1β and Tnf-α were also substantially higher in both colon tissues and BMDMs from *Rnf128*^*−/−*^ mice following DSS and LPS treatment (Fig. 3K, L). Analysis of GEO datasets (GSE92415, GSE38713, GSE51785, GSE59071, GSE73661, and GSE87466) revealed a significant negative correlation between RNF128 expression and proinflammatory cytokines (IL-6, IL-1β, TNF-α, and IL-1α) (Figure S3A–F). Collectively, these data suggest that *Rnf128* deficiency enhances the production of proinflammatory cytokines, including those produced by macrophages.

### *RNF128* deficiency in myeloid cells contributes to the aggravation of DSS-induced colitis

**Fig. 4:**
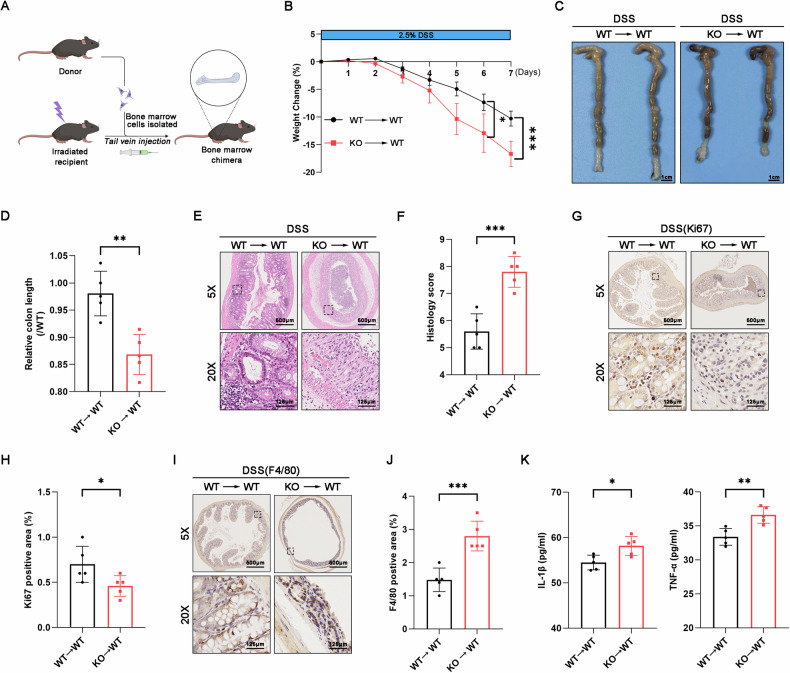
RNF128 in myeloid cells prevents DSS-induced colitis. **A** Schematic diagram of the bone marrow transplantation experiments. **B** *Rnf128*^*+/+*^ → *Rnf128*^*+/+*^ (*n* = 5) and *Rnf128*^*−/−*^ → *Rnf128*^*+/+*^ mice (*n* = 5) were exposed to 2.5% DSS for 7 days. The body weight of mice was measured daily. Statistical data are presented as mean ± SD. *P < 0.05, ***P < 0.001. **C** Representative images of colons from the mice in (**B**). Scale bars, 1 cm. **D** The colon length in (**B**) was measured. Statistical data are presented as mean ± SD. **P < 0.01. **E** Representative H&E-staining images of colon sections from mice in (**B**). Scale bar, 500 μm. **F** Histopathology score in (**E**) were measured. Statistical data are presented as mean ± SD. ***P < 0.001. **G** Representative Ki67 staining images of distal colon tissue sections from the mice in (**B**). Scale bars, 500 μm. **H** The Ki67 positive areas in (**G**) were analyzed. Statistical data are presented as mean ± SD. *P < 0.05. **I** Representative F4/80 staining images of distal colon tissue sections from the mice in (**B**). Scale bar = 500 µm. **J** The F4/80 positive areas in (**I**) were measured. Statistical data are presented as mean ± SD. ***P < 0.001. **K** ELISA analysis of IL-1β and TNF-α levels in serum from the mice in (**B**). Statistical data are presented as mean ± SD. *P < 0.05, **P < 0.01.

Intestinal immune cells, such as macrophages, neutrophils, and dendritic cells (DCs), primarily originate from hematopoietic cells in the bone marrow. We conducted a bone marrow transfer experiment to determine whether *RNF128* deficiency in macrophages contributes to the aggravation of DSS-induced colitis (Fig. 4A). Western blot analysis showed that the bone marrow chimeric mice model was successfully established (Figure S4A). Consistent with the observations in the DSS-treated *Rnf128*^*−/−*^ mice, the *Rnf128*^*+/+*^ mice that received *Rnf128*^*−/−*^ bone marrow (*Rnf128*^*−/−*^ → *Rnf128*^*+/+*^) exhibited a more severe colitis phenotype than the *Rnf128*^*+/+*^ mice that received *Rnf128*^*+/+*^ bone marrow cells (*Rnf128*^*+/+*^→ *Rnf128*^*+/+*^) upon DSS treatment, along with greater body weight loss, shorter colon length, and worsened colonic inflammatory histopathology (Fig. 4B–F). Additionally, the *Rnf128*^*−/−*^→*Rnf128*^*+/+*^ group presented increased macrophage infiltration, fewer Ki67-positive cells and higher levels of proinflammatory cytokines (Il-1β and Tnf-α) in the serum of the mice with colitis than the *Rnf128*^*+/+*^→ *Rnf128*^*+/+*^ group (Fig. 4G–K). Considering previous results that RNF128 is primarily localized in macrophages, we speculate that macrophage RNF128 plays important roles in mediating resistance to DSS-induced colitis. To further investigate the role of RNF128 in macrophages, *Rnf128*^*+/+*^ or *Rnf128*^*−/−*^ mice were intravenously injected with clodronate liposomes. Immunohistochemistry revealed that clodronate liposomes effectively eliminated macrophages (Figure S4B). Depletion of macrophages abolished the difference in the extent of intestinal inflammation between *Rnf128*^*+/+*^ and *Rnf128*^*−/−*^ mice (Figure S4C–F). Overall, these findings indicate that Rnf128 plays a role in suppressing colonic inflammation through macrophages.

### S100A8 is a novel protein that interacts with RNF128

**Fig. 5:**
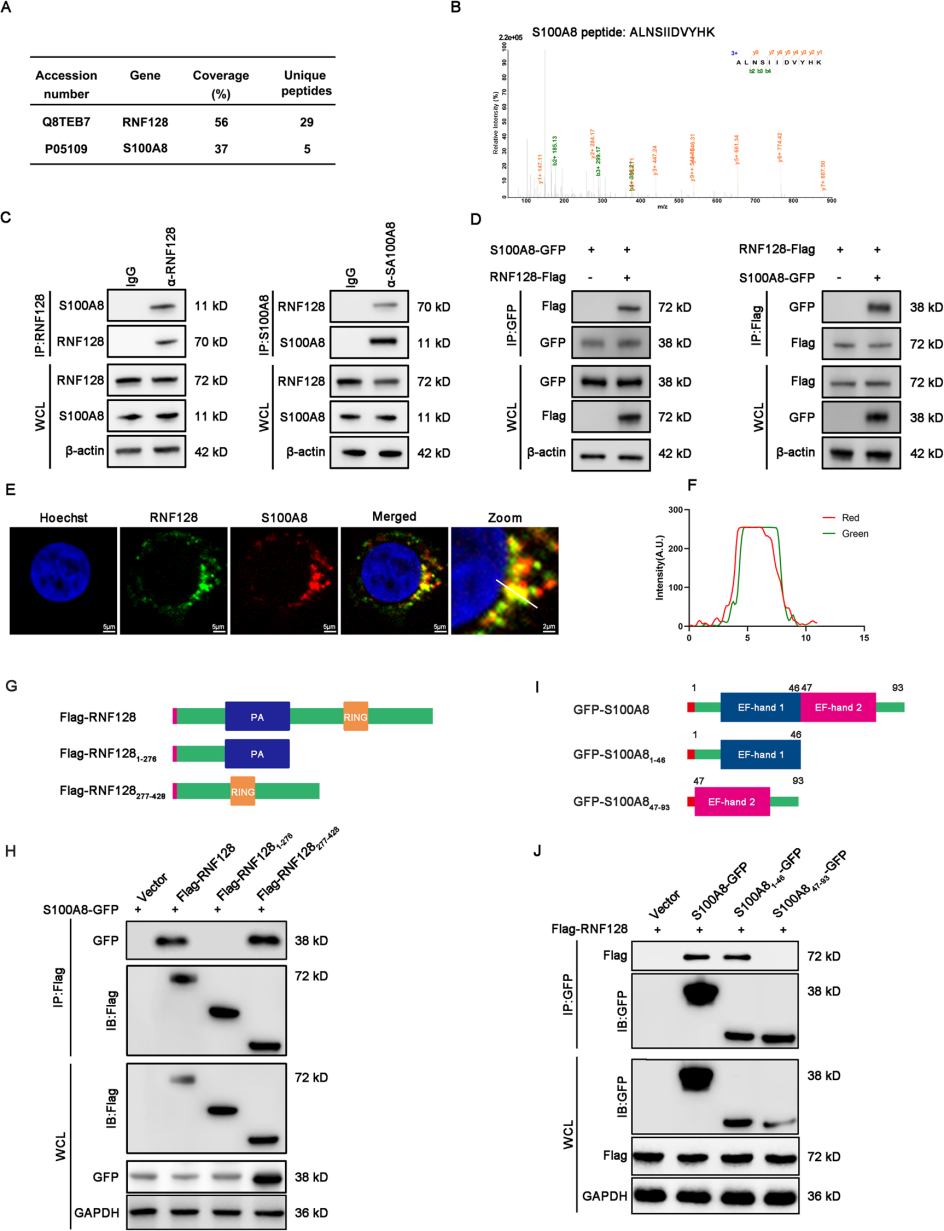
RNF128 interacts with S100A8. **A** THP-1 cells were transfected with empty vector or RNF128-Flag. The cell lysates were immunoprecipitated with an anti-FLAG antibody. S100A8 was identified via mass spectrometry. **B** Mass spectrometry analysis of S100A8 peptide immunoprecipitated by RNF128. **C** Total THP-1 cell lysates were immunoprecipitated with anti-RNF128 or anti-S100A8 antibodies. RNF128 and S100A8 were detected by western blot. **D** 239T cells were co-transfected with RNF128-Flag and S100A8-GFP for 48 h. Total cell lysates were immunoprecipitated with anti-Flag or anti-GFP antibodies. RNF128-Flag and S100A8-GFP were detected by western blot. **E** Immunofluorescence assay of RNF128 and S100A8 in THP-1 cells. Representative confocal microscopy images were shown. Scale bars, 5 μm. **F** The colocalization of RNF128 and S100A8 in THP-1 cells was analyzed. **G** Schematic diagram of RNF128-Flag and its truncation mutants. **H** 239T cells were co-transfected with RNF128-Flag, RNF128-Flag_1-276_, RNF128-Flag_277-428_ and S100A8-GFP for 48 h. Total cell lysates were immunoprecipitated with an anti-Flag antibody. The immunoprecipitation complex was detected with anti-GFP and anti-Flag antibodies. **I** Schematic diagram of S100A8-GFP and its truncation mutants. **J** 239T cells were co-transfected with S100A8-GFP, S100A8-GFP_1-46_, S100A8-GFP_47-93_ and RNF128-Flag for 48 h. Total cell lysates were immunoprecipitated with an anti-GFP antibody. The immunoprecipitation complex was detected by anti-GFP and anti-Flag antibodies.

To identify the underlying molecular mechanism responsible for the protective role of RNF128 in colitis, we performed an immunoprecipitation-coupled mass spectrometry screen. Gene Ontology (GO) analysis revealed that the proteins coimmunoprecipitated with RNF128 were involved mainly in the innate immune response, protein catabolic process, cytokine-mediated signaling pathway and NIK/NF-κB signaling (Figure S5A). The Kyoto Encyclopedia of Genes and Genomes (KEGG) pathway was enriched mainly in the mTOR signaling pathway, TNF signaling pathway, Jak-STAT signaling pathway and inflammatory bowel disease (Figure S5B). Interestingly, S100A8, a gut mucosal damage-associated molecular patterns (DAMP) involved in tissue inflammation, was identified as a potential interacting partner of RNF128 (Fig. 5A, B). Reciprocal co-immunoprecipitation (Co-IP) experiments confirmed the physical interaction between RNF128 and S100A8 (Fig. 5C, D). Moreover, RNF128 and S100A8 exhibited marked colocalization (Fig. 5E, F). RNF128 contains two highly conserved domains, the PA domain (protease-associated domain) and the RING domain (Fig. 5G). We constructed truncated forms of RNF128 (RNF128_1-276_ and RNF128_277-428_) and found that the truncation (RNF128_277-428_) containing the RING domain was able to interact with S100A8 (Fig. 5H). We further generated a series of S100A8 truncations on the basis of its functional domain and performed Co-IP experiments with RNF128. The results revealed that the EF-hand1 domain (residues 1–46) was essential for the interaction with RNF128 (Fig. 5I, J). We used AlphaFold2 to generate a predicted interaction model of S100A8 in complex with RNF128 (Figure S5C–F). These results indicate that the EF-hand1 domain (residues 1–46) of S100A8 and the RING domain of RNF128 are essential for their interaction.

### RNF128 promotes the autophagic degradation of S100A8

**Fig. 6:**
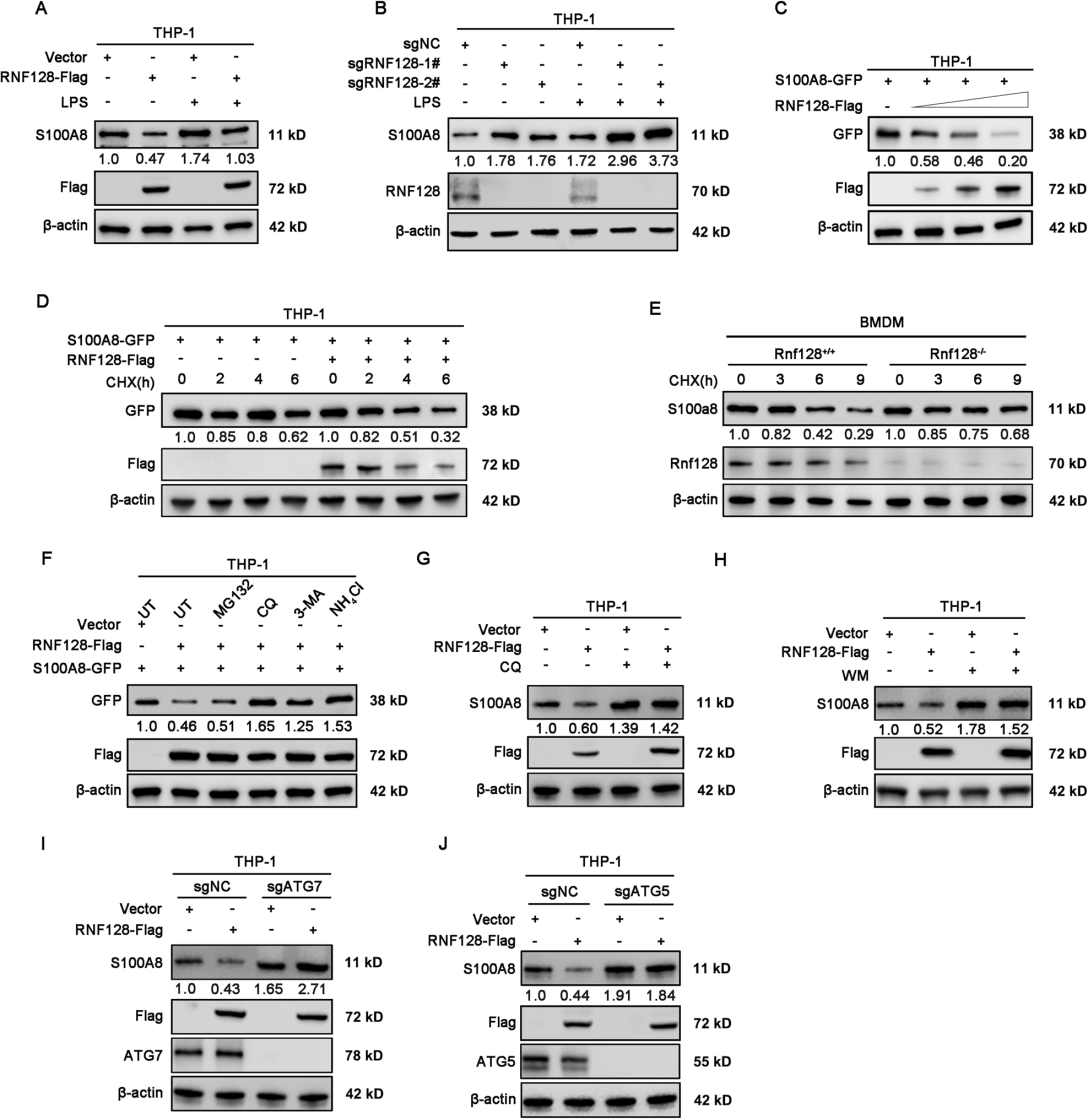
RNF128 promotes the degradation of S100A8 by autophagy. **A** Western blot analysis of S100A8 expression in THP-1 cells stably overexpressing RNF128-Flag treated with or without LPS (200 ng/ml) for 8 h. **B** Western blot analysis of S100A8 expression in stable RNF128-knockout THP-1 cells treated with or without LPS (200 ng/ml) for 8 h. **C** THP-1 cells were co-transfected with S100A8-GFP and increasing concentrations of RNF128-Flag for 48 h. The expression of S100A8-GFP was analyzed by western blot. **D** THP-1 cells stably overexpressing RNF128-Flag were transfected with GFP-S100A8 and incubated with CHX for the indicated time. The expression of S100A8-GFP was analyzed by western blot. **E** *Rnf128*^*+/+*^ and *Rnf128*^*−/−*^ BMDMs were stimulated with LPS (200 µg/ml) for 8 h and subsequently treated with cycloheximide (100 µg/mL) for the indicated time. The expression of S100a8 was detected by western blot. **F** THP-1 cells stably overexpressing RNF128-Flag were transfected with S100A8-GFP and treated with MG132, CQ, 3-MA, or NH_4_Cl. The expression of S100A8-GFP was analyzed via western blot. **G** THP-1 cells stably overexpressing RNF128 were treated with CQ (50 mM) for 4 h. The expression of S100A8 was detected by western blot. **H** THP-1 cells stably overexpressing RNF128 were treated with wortmannin (WM, 10 μM) for 12 h. The expression of S100A8 was detected by western blot. **I** THP-1 cells with stable ATG7 knockout or control cells were transfected with RNF128-Flag for 48 h. The expression of S100A8 was measured by western blot. **J** THP-1 cells with stable ATG5 knockout or control cells were transfected with RNF128-Flag for 48 h. The expression of S100A8 was measured by western blot.

Since RNF128 is an E3 ubiquitin ligase, we wondered whether the protein level of S100A8 is affected by their interaction. Western blot analysis revealed that overexpression of RNF128 downregulated S100A8, whereas knockdown of RNF128 led to the accumulation of S100A8 with or without LPS stimulation (Fig. 6A, B). Transfection of 293T cells with increasing amounts of RNF128 plasmid resulted in a concentration-dependent decrease in S100A8 protein levels (Fig. 6C). The cycloheximide chase assay demonstrated that RNF128 significantly accelerated the degradation of S100A8 (Fig. 6D, E). To determine the degradation pathways of S100A8 upon RNF128 overexpression, we treated control or RNF128-overexpressing cells with a proteasome inhibitor (MG132) or autophagy inhibitors (3MA, Wortmannin, CQ or NH_4_Cl). The results showed that lysosomal inhibition, but not proteasomal inhibition, substantially blocked the degradation of S100A8 (Fig. 6F–H). Moreover, inhibiting autophagy via ATG5, ATG7 knockout or BECN1, ATG12 knockdown also reversed RNF128’s negative regulation of S100A8 (Fig. 6I, J, Figure S6A, B). These data suggest that RNF128 interacts with S100A8 and promotes its autophagic degradation.

### Tollip mediates the autophagic degradation of S100A8 regulated by RNF128

**Fig. 7:**
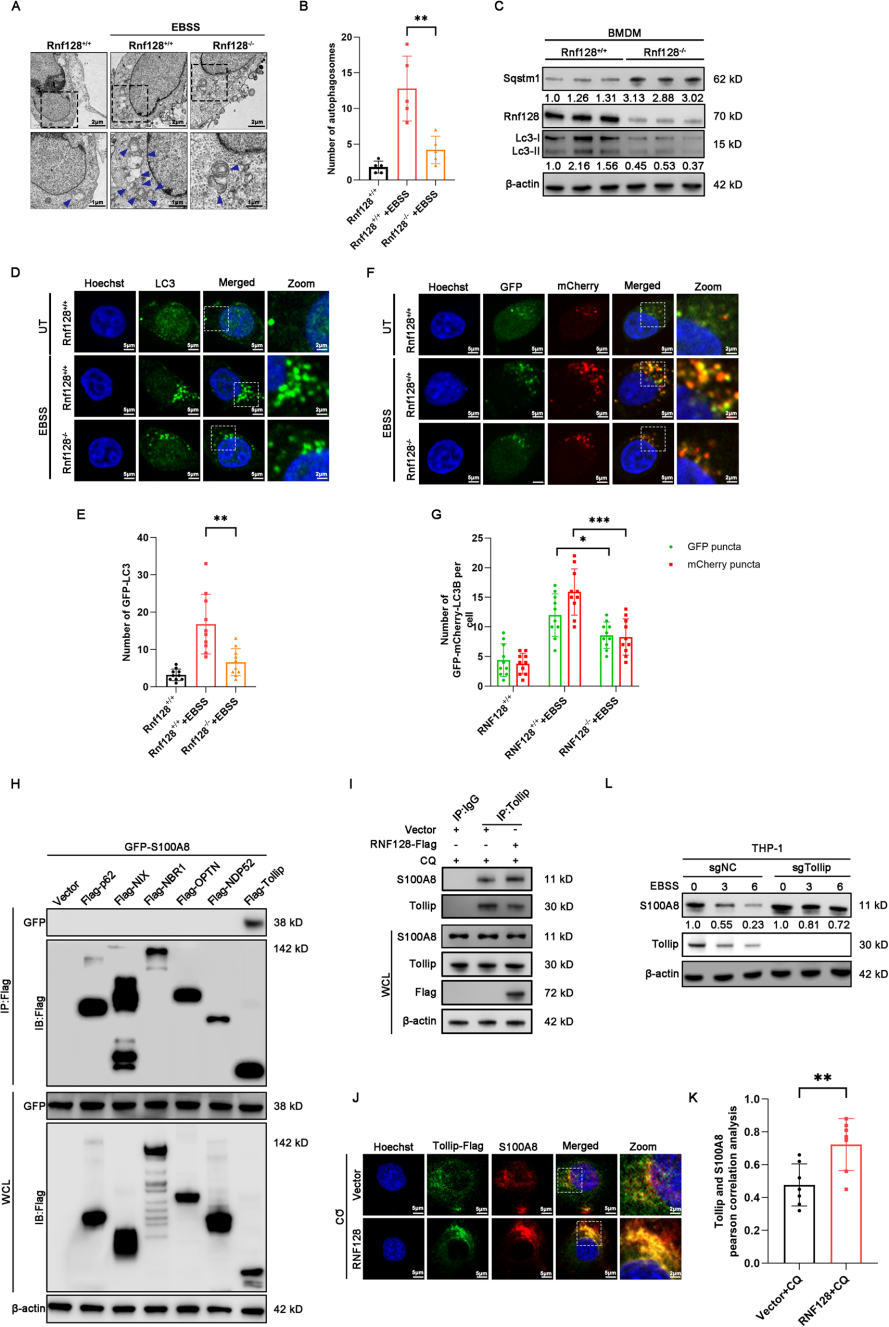
RNF128 promotes Tollip-mediated selective autophagic degradation of S100A8. **A** Representative TEM images of *Rnf128*^*+/+*^ and *Rnf128*^*–/–*^ BMDMs treated with or without EBSS for 2 h. The blue arrows indicate autophagic structures. Scale bars = 2 µm. **B** The number of autophagosomes per cell in (**A**) was quantified. The data are shown as mean ± SD. Five cells were counted. **P < 0.01. **C** Western blot analysis of LC3 and Sqstm1/p62 protein levels in BMDMs from *Rnf128*^*+/+*^ and *Rnf128*^*–/–*^ mice stimulated with LPS (200 µg/ml) for 8 h. **D** *Rnf128*^*+/+*^ and *Rnf128*^*–/–*^ BMDMs were transfected with GFP-LC3B for 48 h. GFP-LC3B distribution was observed by confocal microscopy. Scale bar, 5 μm. **E** The number of autophagosomes per cell in (**D**) was quantified. Data shown as mean ± SD. Ten cells were counted. Statistical data are presented as mean ± SD. **P < 0.01. **F** *Rnf128*^*+/+*^ and *Rnf128*^*–/–*^ BMDMs were transfected with GFP-mCherry-LC3B for 48 h. GFP-mCherry-LC3B distribution was observed via confocal microscopy. Scale bar, 5 μm. **G** The numbers of autophagosomes and autolysosomes per cell in (**F**) were quantified. The data are shown as mean ± SD. Ten cells were counted. Statistical data are presented as mean ± SD. *P < 0.05; ***P < 0.001. **H** 293T cells were co-transfected with the indicated autophagy cargo receptors and S100A8-GFP for 48 h. Total cell lysates were immunoprecipitated with anti-Flag antibody. The immunoprecipitation complex was detected with anti-GFP and anti-Flag antibodies. **I** RNF128-Flag stably overexpressing or control THP-1 cells were treated with CQ (50 μM) for 4 h, and total cell lysates were immunoprecipitated with an anti-Tollip antibody. S100A8 and Tollip were detected by western blot. **J** THP-1 cells stably overexpressing RNF128 were transfected with Flag-Tollip and treated with CQ (50 μM) for 4 h. The co-localization of Flag-Tollip and S100A8 was observed by confocal microscopy. Representative confocal microscopy images were shown. Scale bars, 5 μm. **K** The colocalization Pearson correlation of Tollip-Flag and S100A8 in (**J**) was quantified. Statistical data are presented as mean ± SD. **P < 0.01. **L** Tollip stable knockout THP-1 or control cells were treated with EBSS for the indicated time, and the expression of S100A8 was detected by western blot.

Next, we investigated the role of RNF128 in regulating autophagy. Transmission electron microscopy (TEM) analysis showed that compared with control cells, *Rnf128*^*−/−*^ BMDM cells exhibited fewer autophagosome-like structures upon EBSS treatment (Fig. 7A, B). Consistently, Rnf128 knockout reduced the number of GFP-LC3B puncta, inhibited the accumulation of endogenous LC3B-II and increased the protein levels of SQSTM1/p62 (Fig. 7C–E). The GFP-mCherry-LC3B plasmid is a useful tool for assessing autophagy flux, as it can label autophagosomes and autophagosomes-lysosomes with red and yellow fluorescence simultaneously [18]. *Rnf128* knockout decreased the number of both autophagosomes and autophagosomes-lysosomes in BMDM cells (Fig. 7F, G). Collectively, these results indicate that RNF128 knockout suppressed the autophagy process.

The selective autophagic degradation of proteins is typically mediated by specific autophagy cargo receptors [19]. To determine which receptor is involved in the degradation of S100A8, we conducted co-immunoprecipitation (Co-IP) experiments in THP-1 cells co-transfected with GFP-S100A8 and several known autophagy cargo receptors. Among these receptors, only Tollip showed a significant interaction with S100A8, implying that Tollip may mediate the selective autophagic degradation of S100A8 (Fig. 7H). Furthermore, RNF128 overexpression increased the colocalization and interaction between S100A8 and Tollip (Fig. 7I–K). Additionally, knockdown of Tollip rescued the decreased S100A8 protein levels induced by EBSS (Fig. 7L). These findings indicate that RNF128 overexpression enhances the interaction between S100A8 and Tollip, thereby promoting the selective autophagic degradation of S100A8.

### RNF128 promotes the ubiquitination of S100A8 at its lysine 36 residue

Before being recognized by autophagy receptors, cargo proteins destined for selective autophagy are often subjected to ubiquitination [20]. RNF128 overexpression significantly promoted the ubiquitination of S100A8, whereas *RNF128* deficiency had an opposite effect (Figure S7A–C). Using ubiquitin mutants (K6, K11, K27, K33, K48, or K63), in which the indicated lysine is retained and all other lysines are replaced with arginine, we found that RNF128 specifically enhanced the K63-linked ubiquitination of S100A8 (Figure S7D). We analyzed the amino acid sequence of S100A8_1-46_, which is essential for the interaction with RNF128, and identified 2 conserved lysines (K7 and K36) (Figure S7E). To determine whether K7 or K36 is the ubiquitination site influenced by RNF128, we constructed S100A8 K-to-R mutated plasmids and found that RNF128 promoted the ubiquitination and degradation of S100A8^WT^ and S100A8^K7R^ but not S100A8^K36R^ (Figure S7F, G). These results indicate that RNF128 facilitates the ubiquitination of S100A8 at its lysine 36 residue.

### Neutralizing antibodies against S100A8 attenuated acute colitis in DSS-treated *Rnf128*^*−/−*^ mice

**Fig. 8:**
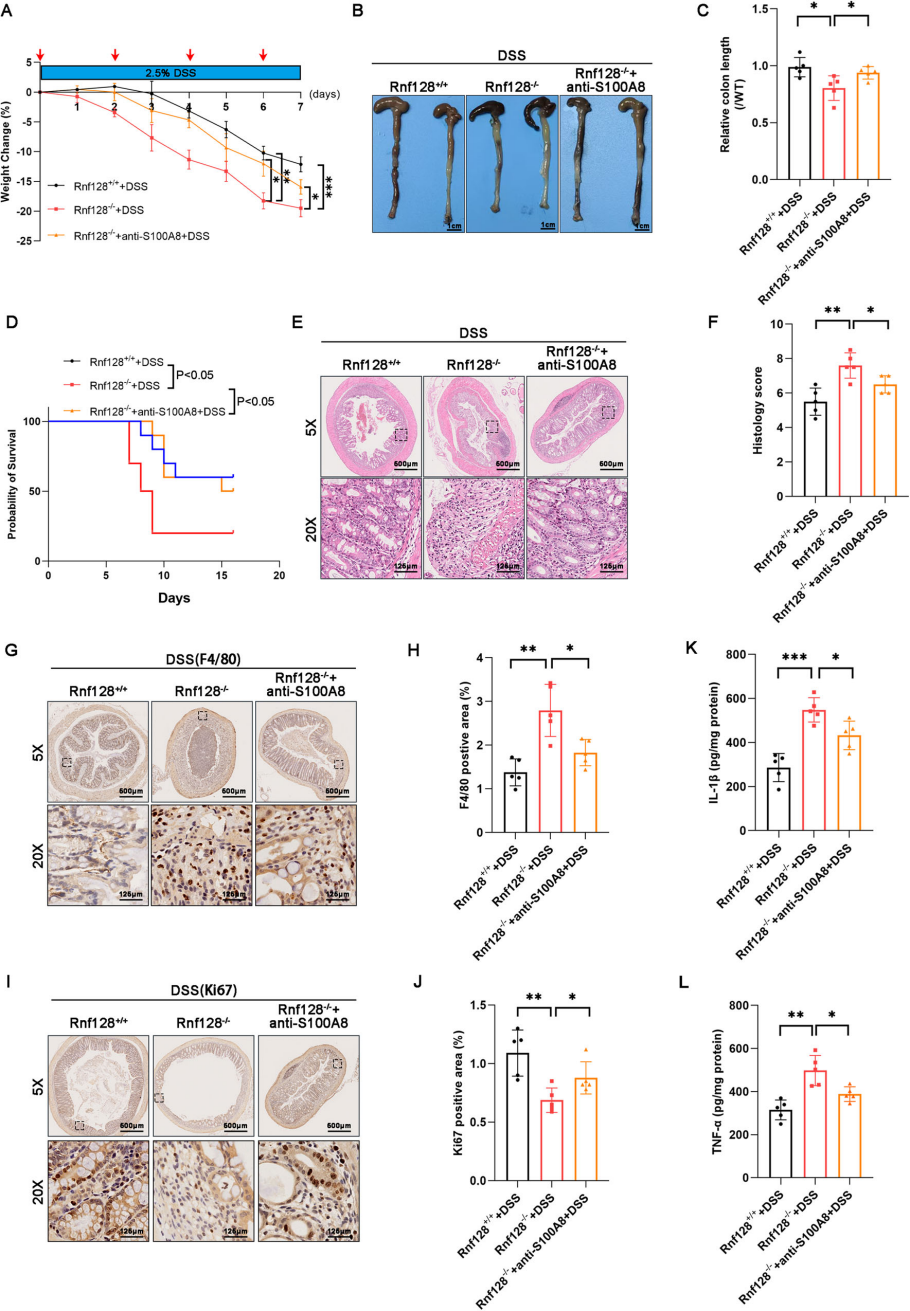
Neutralizing antibodies to S100A8 attenuate DSS-induced colonic injury in *Rnf128*^*−/−*^ mice. **A** *Rnf128*^*+/+*^ (*n* = 5) and *Rnf128*^*−/−*^ (*n* = 5) mice treated with 2.5% DSS were treated with an anti-S100A8 neutralizing antibody (200 μg, i.p.) on Days 0, 2, 4, and 6. Body weight of mice were measured daily. Statistical data are presented as mean ± SD. *P < 0.05; **P < 0.01; ***P < 0.001. **B** Representative images of colons from the mice in (**A**). Scale bars, 1 cm. **C** The colon length in (**B**) was measured and analyzed. *P < 0.05. **D** The survival of *Rnf128*^*+/+*^ (*n* = 10) and *Rnf128*^*−/−*^ (*n* = 10) mice treated with 3% DSS and an anti-S100A8 neutralizing antibody. **E** Representative images of H&E-stained colon sections from the mice in (**A**). Scale bar: 500 μm. **F** Histopathology score in (**E**) was measured (*n* = 5). Statistical data are presented as mean ± SD. *P < 0.05; **P < 0.01. **G** Representative F4/80 staining images of colon sections from the mice in (**A**). Scale bars = 500 μm. **H** The F4/80 positive areas in (**G**) were measured and analyzed. Statistical data are presented as mean ± SD. *P < 0.05; **P < 0.01. **I** Representative images of Ki67 staining in cross-sections of distal colon tissues from the mice in (**A**). Scale bars = 500 μm. **J** Ki67 positive areas were measured and analyzed (*n* = 5). Statistical data are presented as mean ± SD. *P < 0.05; **P < 0.01. **K**, **L** ELISA analysis of IL-1β (**I**) and TNF-α (**K**) levels in the colon tissue from the mice in (**A**). Statistical data are presented as mean ± SD. *P < 0.05; **P < 0.01; ***P < 0.001.

To determine whether elevated S100A8 expression contributes to the severe colitis phenotype in *Rnf128*^*−/−*^ mice, we blocked S100A8 with its neutralizing antibodies in the DSS-induced colitis model. Intraperitoneal injection of S100A8-neutralizing antibodies not only significantly ameliorated body weight loss and colon shortening but also improved the survival of DSS-treated *Rnf128*^*−/−*^ mice (Fig. 8A–D). H&E and Histological analysis showed that administration of the S100A8-neutralizing antibody also improved DSS-induced colon damage, reduced macrophage infiltration and increased the number of Ki67-positive cells in *Rnf128*^*−/−*^ mice (Fig. 8E–J). In addition, the levels of proinflammatory cytokines (Il-1β and Tnf-α) were substantially reduced by S100A8-neutralizing antibody treatment (Fig. 8K, L). Taken together, these data indicate that treatment with S100A8-neutralizing antibodies improves colitis in DSS-treated *Rnf128*^*−/−*^ mice.

## Discussion

Excessive activation of the immune response is a hallmark of IBD [21]. Macrophages, although playing crucial roles in maintaining intestinal homeostasis, may act as “perpetrators” in the initiation and worsening of colitis [22]. Fine-tuning macrophages is considered an important approach to prevent inflammation [4, 23, 24]. Several studies have explored the upstream regulatory factors and associated mechanisms involved in macrophage activation during the progression of IBD. For example, YTHDC1 in macrophages enhanced the integrity of colonic epithelial barrier and improved IBD severity by promoting Rhoh and Nme1 expression in an m6A-dependent manner [25]. The deubiquitinase OTUD6A in macrophages exacerbates intestinal inflammation and colitis by activating the NLRP3 inflammasome [26]. Herein, we identified the anti-inflammatory function of macrophage RNF128 in IBD progression. RNF128 is downregulated in proinflammatory macrophages, and decreased RNF128 leads to elevated levels of effector cytokines, thus accelerating IBD development. Interestingly, a recent study reported that *RNF128* deficiency in intestinal epithelial cells promoted colitis and colorectal tumorigenesis by interacting with IL-6Rα and glycoprotein gp130 [13, 15]. Thus, we conclude that RNF128 from different cell sources plays a protective role in colitis. Although our study demonstrated the protective role of macrophage RNF128 in colitis and its underlying molecular mechanism, the reasons for the reduced expression of RNF128 in macrophages during colitis remain unclear, suggesting a novel research direction.

S100A8, a member of S100 protein family, is expressed predominantly in neutrophils and monocytes and has been implicated in various inflammatory diseases, including IBD [27]. As a damage-associated molecular patterns (DAMP), S100A8 exert pro- or anti-inflammatory effects under different diseases conditions [28, 29]. For example, S100A8 could stimulate the migration of neutrophils to inflammatory sites, thereby triggering inflammatory responses. Conversely, S100A8 could alleviate asthma symptoms by modulating mast cells activation and eosinophil recruitment [30]. Although the functional roles of S100A8 in inflammation have been extensively studied, the mechanisms regulating its activity during intestinal inflammation are still largely unknown. Here, we identified the ubiquitin ligase RNF128 as an upstream regulatory molecule of S100A8. RNF128 interferes with S100A8 and decreases its protein stability through autophagy. S100A8 is characterized by two EF-hand domains (EF-hand 1 and EF-hand 2) with different affinities for calcium [31]. Our findings indicate that the EF-hand 1 domain of S100A8 is primarily responsible for its interaction with RNF128. Given the colitis-promoting effects of S100A8, targeting S100A8 may be an attractive therapeutic strategy for IBD. Previous studies have reported that S100A8-neutralizing antibodies or delivery of S100A8 inhibitors via nanomaterials could attenuate the inflammatory response and enhance the therapeutic efficacy of ulcerative colitis treatment [32, 33]. Consistent with these findings, our data demonstrate that neutralizing antibodies against S100A8 improve colitis damage in DSS-treated *Rnf128*^*−/−*^ mice, highlighting the importance of the RNF128‒S100A8 axis in macrophages for the development of therapeutic approaches for IBD.

Selective autophagy involves the recognition and targeting of specific cargo, such as damaged organelles, misfolded proteins, or invading pathogens, for lysosomal degradation [34]. This process is typically mediated by specific autophagy receptors [35]. Impaired autophagy is usually linked to the exacerbation of intestinal inflammation [36]. Loss of the autophagy-related gene Atg16l1 inhibited the degradation of TRIF mediated by autophagy receptors SQSTM1 and Tax1BP1, thus driving TRIF-dependent inflammatory signaling in S. Typhimurium-induced ileitis [37]. p62/SQSTM1 targets claudin-2 for selective autophagy in stressed intestinal epithelium and protects mice from DSS-induced colitis [38]. Our work revealed a novel pathway between autophagy and intestinal inflammation: *RNF128* deficiency in macrophages suppresses the autophagic degradation of S100A8 mediated by the cargo receptor Tollip. The upregulated S100A8 increased the secretion of inflammatory factors and promoted colonic inflammation. Our study also helps to explain previous findings that overexpressing Tollip in the intestinal epithelium protected mice from DSS-induced colitis, whereas Tollip-KO mice develop severe colitis [39]. Ubiquitination marks proteins for degradation, including autophagy-mediated targeting [40]. Accordingly, our analysis determined that S100A8 associates primarily with the K63-linked ubiquitin at K36 under our experimental conditions. Our study provides new insights into the interaction between the autophagic degradation and ubiquitination of S100A8. However, the exact mechanisms by which RNF128 regulates autophagy are still unclear and require further investigation.

In summary, we demonstrated for the first time that macrophage *RNF128* deficiency promotes colitis progression by suppressing Tollip-mediated autophagic degradation of S100A8. Our findings underscore the protective roles of RNF128 in colitis and contribute to the understanding of S100A8 regulation. This novel RNF128-Tollip-S100A8 axis may provide novel insights into new targets for IBD therapy.

## Supplementary information

Supplementary Materials

Original image for western blot

## Data Availability

The data are available in the article and obtained from the corresponding author upon reasonable request.

## References

[1] Neurath MF. Targeting immune cell circuits and trafficking in inflammatory bowel disease. Nat Immunol. 2019;20:970–9. 31235952 10.1038/s41590-019-0415-0

[2] Adolph TE, Zhang J. Diet fuelling inflammatory bowel diseases: preclinical and clinical concepts. Gut. 2022;71:2574–86. 36113981 10.1136/gutjnl-2021-326575 PMC9664119

[3] Wijnands AM, Penning de Vries BBL, Lutgens M, Bakhshi Z, Al Bakir I, Beaugerie L, et al. Dynamic prediction of advanced colorectal neoplasia in inflammatory bowel disease. Clin Gastroenterol Hepatol. 2024;22:1697–708. 38431223 10.1016/j.cgh.2024.02.014

[4] Ma S, Zhang J, Liu H, Li S, Wang Q. The role of tissue-resident macrophages in the development and treatment of inflammatory bowel disease. Front Cell Dev Biol. 2022;10:896591. 35721513 10.3389/fcell.2022.896591 PMC9199005

[5] Hou Q, Huang J, Ayansola H, Masatoshi H, Zhang B. Intestinal stem cells and immune cell relationships: potential therapeutic targets for inflammatory bowel diseases. Front Immunol. 2020;11:623691. 33584726 10.3389/fimmu.2020.623691 PMC7874163

[6] Li Q, Lin L, Zhang C, Zhang H, Ma Y, Qian H, et al. The progression of inorganic nanoparticles and natural products for inflammatory bowel disease. J Nanobiotechnology. 2024;22:17. 38172992 10.1186/s12951-023-02246-x PMC10763270

[7] Hegarty LM, Jones GR, Bain CC. Macrophages in intestinal homeostasis and inflammatory bowel disease. Nat Rev Gastroenterol Hepatol. 2023;20:538–53. 37069320 10.1038/s41575-023-00769-0

[8] Na YR, Stakenborg M, Seok SH, Matteoli G. Macrophages in intestinal inflammation and resolution: a potential therapeutic target in IBD. Nat Rev Gastroenterol Hepatol. 2019;16:531–43. 31312042 10.1038/s41575-019-0172-4

[9] Whiting CC, Su LL, Lin JT, Fathman CG. GRAIL: a unique mediator of CD4 T-lymphocyte unresponsiveness. FEBS J. 2011;278:47–58. 21078124 10.1111/j.1742-4658.2010.07922.x PMC3058357

[10] Martinho MS, Nancarrow DJ, Lawrence TS, Beer DG, Ray D. Chaperones and ubiquitin ligases balance mutant p53 protein stability in esophageal and other digestive cancers. Cell Mol Gastroenterol Hepatol. 2021;11:449–64. 33130332 10.1016/j.jcmgh.2020.10.012 PMC7788241

[11] Liu PY, Chen CY, Lin YL, Lin CM, Tsai WC, Tsai YL, et al. RNF128 regulates neutrophil infiltration and myeloperoxidase functions to prevent acute lung injury. Cell Death Dis. 2023;14:369. 37344492 10.1038/s41419-023-05890-1 PMC10284794

[12] Haymaker C, Yang Y, Wang J, Zou Q, Sahoo A, Alekseev A, et al. Absence of Grail promotes CD8(+) T cell anti-tumour activity. Nat Commun. 2017;8:239. 28798332 10.1038/s41467-017-00252-w PMC5552797

[13] Mukai A, Iijima H, Hiyama S, Fujii H, Shinzaki S, Inoue T, et al. Regulation of anergy-related ubiquitin E3 ligase, GRAIL, in murine models of colitis and patients with Crohn’s disease. J Gastroenterol. 2014;49:1524–35. 24356810 10.1007/s00535-013-0923-x

[14] Egawa S, Iijima H, Shinzaki S, Nakajima S, Wang J, Kondo J, et al. Upregulation of GRAIL is associated with remission of ulcerative colitis. Am J Physiol Gastrointest Liver Physiol. 2008;295:G163–9. 18467499 10.1152/ajpgi.90242.2008

[15] He TS, Cai K, Lai W, Yu J, Qing F, Shen A, et al. E3 ubiquitin ligase RNF128 attenuates colitis and colorectal tumorigenesis by triggering the degradation of IL-6 receptors. J Adv Res. 2024;24:00262-5.10.1016/j.jare.2024.06.02538964734

[16] Liu X, Luo W, Chen J, Hu C, Mutsinze RN, Wang X, et al. USP25 deficiency exacerbates acute pancreatitis via up-regulating TBK1-NF-kappaB signaling in macrophages. Cell Mol Gastroenterol Hepatol. 2022;14:1103–22. 35934222 10.1016/j.jcmgh.2022.07.013 PMC9490099

[17] Neurath MF. Targeting cytokines in inflammatory bowel disease. Sci Transl Med. 2022;14:eabq4473. 36516267 10.1126/scitranslmed.abq4473

[18] Kimura S, Noda T, Yoshimori T. Dissection of the autophagosome maturation process by a novel reporter protein, tandem fluorescent-tagged LC3. Autophagy. 2007;3:452–60. 17534139 10.4161/auto.4451

[19] Liu J, Wu Y, Meng S, Xu P, Li S, Li Y, et al. Selective autophagy in cancer: mechanisms, therapeutic implications, and future perspectives. Mol Cancer. 2024;23:22. 38262996 10.1186/s12943-024-01934-y PMC10807193

[20] Kirkin V, Lamark T, Sou YS, Bjorkoy G, Nunn JL, Bruun JA, et al. A role for NBR1 in autophagosomal degradation of ubiquitinated substrates. Mol Cell. 2009;33:505–16. 19250911 10.1016/j.molcel.2009.01.020

[21] Zhang K, Guo J, Yan W, Xu L. Macrophage polarization in inflammatory bowel disease. Cell Commun Signal. 2023;21:367. 38129886 10.1186/s12964-023-01386-9 PMC10734116

[22] Du Y, Rong L, Cong Y, Shen L, Zhang N, Wang B. Macrophage polarization: an effective approach to targeted therapy of inflammatory bowel disease. Expert Opin Ther Targets. 2021;25:191–209. 33682588 10.1080/14728222.2021.1901079

[23] Wang EJ, Wu MY, Ren ZY, Zheng Y, Ye RD, Tan CSH, et al. Targeting macrophage autophagy for inflammation resolution and tissue repair in inflammatory bowel disease. Burns Trauma. 2023;11:tkad004. 37152076 10.1093/burnst/tkad004 PMC10157272

[24] Pan X, Zhu Q, Pan LL, Sun J. Macrophage immunometabolism in inflammatory bowel diseases: From pathogenesis to therapy. Pharmacol Ther. 2022;238:108176. 35346728 10.1016/j.pharmthera.2022.108176

[25] Ge X, Xue G, Ding Y, Li R, Hu K, Xu T, et al. The loss of YTHDC1 in gut macrophages exacerbates inflammatory bowel disease. Adv Sci. 2023;10:e2205620. 10.1002/advs.202205620 PMC10190588 36922750

[26] Liu X, Fang Y, Lv X, Hu C, Chen G, Zhang L, et al. Deubiquitinase OTUD6A in macrophages promotes intestinal inflammation and colitis via deubiquitination of NLRP3. Cell Death Differ. 2023;30:1457–71. 36932155 10.1038/s41418-023-01148-7 PMC10244424

[27] Casavant E, Park KT, Elias JE. Proteomic discovery of stool protein biomarkers for distinguishing pediatric inflammatory bowel disease flares. Clin Gastroenterol Hepatol. 2020;18:2618–9.e2611. 31499250 10.1016/j.cgh.2019.08.052

[28] Vogl T, Stratis A, Wixler V, Voller T, Thurainayagam S, Jorch SK, et al. Autoinhibitory regulation of S100A8/S100A9 alarmin activity locally restricts sterile inflammation. J Clin Invest. 2018;128:1852–66. 29611822 10.1172/JCI89867 PMC5919817

[29] Boyapati RK, Rossi AG, Satsangi J, Ho GT. Gut mucosal DAMPs in IBD: from mechanisms to therapeutic implications. Mucosal Immunol. 2016;9:567–82. 26931062 10.1038/mi.2016.14

[30] Zhao J, Endoh I, Hsu K, Tedla N, Endoh Y, Geczy CL. S100A8 modulates mast cell function and suppresses eosinophil migration in acute asthma. Antioxid Redox Signal. 2011;14:1589–1600. 21142608 10.1089/ars.2010.3583

[31] Hayden JA, Brophy MB, Cunden LS, Nolan EM. High-affinity manganese coordination by human calprotectin is calcium-dependent and requires the histidine-rich site formed at the dimer interface. J Am Chem Soc. 2013;135:775–87. 23276281 10.1021/ja3096416 PMC3575579

[32] Fujita Y, Khateb A, Li Y, Tinoco R, Zhang T, Bar-Yoseph H, et al. Regulation of S100A8 stability by RNF5 in intestinal epithelial cells determines intestinal inflammation and severity of colitis. Cell Rep. 2018;24:3296–311.e3296. 30232010 10.1016/j.celrep.2018.08.057 PMC6185744

[33] Su F, Ye W, Shen Y, Xie Y, Zhang C, Zhang Q, et al. Immuno-nanocomplexes target heterogenous network of inflammation and immunity in myocardial infarction. Adv Sci. 2024;11:e2402267. 10.1002/advs.202402267 PMC11423151 39049710

[34] Vargas JNS, Hamasaki M, Kawabata T, Youle RJ, Yoshimori T. The mechanisms and roles of selective autophagy in mammals. Nat Rev Mol Cell Biol. 2023;24:167–85. 36302887 10.1038/s41580-022-00542-2

[35] Lamark T, Johansen T. Mechanisms of selective autophagy. Annu Rev Cell Dev Biol. 2021;37:143–69. 34152791 10.1146/annurev-cellbio-120219-035530

[36] Levine B, Mizushima N, Virgin HW. Autophagy in immunity and inflammation. Nature. 2011;469:323–35. 21248839 10.1038/nature09782 PMC3131688

[37] Samie M, Lim J, Verschueren E, Baughman JM, Peng I, Wong A, et al. Selective autophagy of the adaptor TRIF regulates innate inflammatory signaling. Nat Immunol. 2018;19:246–54. 29358708 10.1038/s41590-017-0042-6

[38] Ahmad R, Kumar B, Tamang RL, Talmon GA, Dhawan P, Singh AB. P62/SQSTM1 binds with claudin-2 to target for selective autophagy in stressed intestinal epithelium. Commun Biol. 2023;6:740. 37460613 10.1038/s42003-023-05116-2 PMC10352296

[39] Maillard MH, Bega H, Uhlig HH, Barnich N, Grandjean T, Chamaillard M, et al. Toll-interacting protein modulates colitis susceptibility in mice. Inflamm Bowel Dis. 2014;20:660–70. 24572204 10.1097/MIB.0000000000000006

[40] Ahsan N, Shariq M, Surolia A, Raj R, Khan MF, Kumar P. Multipronged regulation of autophagy and apoptosis: emerging role of TRIM proteins. Cell Mol Biol Lett. 2024;29:13. 38225560 10.1186/s11658-023-00528-8 PMC10790450

