# Supplementary material for: RNF128 deficiency in macrophages promotes colonic inflammation by suppressing the autophagic degradation of S100A8
Source: Cell Death Dis. 2025 Jan 15;16(1):20. doi: 10.1038/s41419-025-07338-0 (PMC11733159; doi:10.1038/s41419-025-07338-0)
Supplement: Supplementary file 1 — Supplementary Materials [file 41419_2025_7338_MOESM1_ESM.docx]

Supplementary Materials

**Table S1 The details of reagents and antibodies used in this study**

| **REAGENT or RESOURCE** | **SOURCE** | **IDENTIFIER** |
| --- | --- | --- |
| **Chemicals, Peptides, and Recombinant Proteins** | | |
| Phorbol 12-myristate 13-acetate (PMA) | MedChemExpress | Cat# HY-18739 |
| Lipopolysaccharides (LPS) | MedChemExpress | Cat# HY-D1056 |
| MG132 | MedChemExpress | Cat# HY-13259 |
| Clodronate Liposomes | Yeasen | Cat# 40337ES08 |
| 3-Methyladenine (3-MA) | MedChemExpress | Cat# HY-19312 |
| Chloroquine | MedChemExpress | Cat# HY-17589A |
| NH4Cl | MedChemExpress | Cat# HY-Y1269 |
| Wortmannin | MedChemExpress | Cat# HY-10197 |
| Thioglycolate sodium | MedChemExpress | Cat# HY-W115724 |
| Protease inhibitor cocktail | MedChemExpress | Cat# HY-K0012 |
| 2,6,4-trinitrobenzene sulfonic acid | Sigma-Aldrich | Cat# P2297 |
| M-CSF | PeproTech | Cat# 315-02 |
| Dextran sulfate sodium (DSS) | MP Biomedicals | MFCD00081551 |
| Lipofectamine 3000 | Invitrogen | Cat# L3000015 |
| Puromycin | Selleck | Cat# S7417 |
| Mouse TNF-α ELISA Kit | Boster | Cat# EK0527 |
| Mouse IL-1β ELISA Kit | Boster | Cat# EK0394 |
| BCA Protein Assay Kit | Beyotime | Cat# P0010S |
| RIPA buffer for Western and IP | Beyotime | Cat# P0013 |
| Fetal bovine serum | Gibco | Cat# A5670701 |
| Alcian Blue Periodic Acid Schiff (AB-PAS) Stain Kit | Solarbio | Cat# G1285 |
| Hematoxylin-Eosin (HE) Stain Kit | Solarbio | Cat# G1120 |
| Hoechst33342(Hoechst) | Polysciences | Cat# BLI894A |
| Protein A/G Agarose | Bio-linkedin | Cat# L-1008 |
| RNAiso | Takara | Cat# 9109 |
| Maxima First Strand cDNA Synthesis Kit | Thermofisher | Cat# K1642 |
| PowerTrack™SYBR Green Master Mix | Thermofisher | Cat# A46111 |
| **Antibodies** | | |
| Mouse monoclonal IgG | Santa Cruz Biotechnology | Cat# sc-2025; RRID: AB_737182 |
| Rabbit polyclonal Tollip | Santa Cruz Biotechnology | Cat# sc-59720; RRID: AB_2303699 |
| Rabbit monoclonal IgG | Proteintech Group | Cat# 30000-0-AP; RRID: AB_2819035 |
| Beclin 1 Polyclonal antibody | Proteintech Group | Cat# 11306-1-AP |
| ATG12 Polyclonal antibody | Proteintech Group | Cat# 11264-1-AP |
| Anti -Ly6g Rabbit pAb | Servicebio | Cat# GB11229 |
| Anti-Ly6C Rabbit pAb | Servicebio | Cat# GB115601 |
| Ly-6G Rabbit mAb | Cell Signaling Technology | Cat# 87048 |
| Anti-LY6C antibody | Abcam | Cat# ab317272 |
| Mouse monoclonal GAPDH | Proteintech Group | Cat#60004-1-Ig; RRID: AB_2107436 |
| Mouse monoclonal β-actin | Proteintech Group | Cat#66009-1-Ig; RRID: AB_2687938 |
| Mouse monoclonal GFP-tag | Proteintech Group | Cat# 66002-1-Ig; RRID: AB_11182611 |
| Rabbit polyclonal GFP-tag | Proteintech Group | Cat# 50430-2-AP, RRID: AB_11042881 |
| Rabbit polyclonal Myc-tag | Proteintech Group | Cat#16286-1-AP; RRID: AB_11182162 |
| Mouse Monoclonal Myc-tag | Proteintech Group | Cat# 60003-2-Ig, RRID: AB_2734122 |
| Mouse Monoclonal Flag-tag | Proteintech Group | Cat# 66008-4-Ig; RRID: AB_2918475 |
| Rabbit polyclonal HA-tag | Proteintech Group | Cat# 51064-2-AP; RRID: AB_11042321 |
| Mouse polyclonal HA-tag | Proteintech Group | Cat# 66006-1-Ig, RRID: AB_2857911 |
| Rabbit polyclonal CD31 | Proteintech Group | Cat# 28083-1-AP; RRID: AB_2881055 |
| Rabbit polyclonal MPO | Proteintech Group | Cat# 22225-1-AP; RRID: AB_2879037 |
| Rabbit polyclonal Tollip | Proteintech Group | Cat# 11315-1-AP; RRID: AB_2256373 |
| Rabbit polyclonal p62 | Proteintech Group | Cat# 18420-1-AP; RRID: AB_10694431 |
| Rabbit polyclonal LC3 | Proteintech Group | Cat# 14600-1-AP; RRID: AB_2137737 |
| Rabbit polyclonal ATG7 | Proteintech Group | Cat# 10088-2-AP; RRID: AB_2062351 |
| Rabbit polyclonal ATG5 | Proteintech Group | Cat# 10181-2-AP; RRID: AB_2062045 |
| Mouse monoclonal S100A8 | Proteintech Group | Cat# 66853-1-Ig; RRID: AB_2882193 |
| Rabbit polyclonal Ki67 | Proteintech Group | Cat# 27309-1-AP; RRID: AB_2756525 |
| Rabbit polyclonal CD11c | Cell Signaling Technology | Cat# 97585; RRID: AB_2800282 |
| Rabbit polyclonal F4/80 | Cell Signaling Technology | Cat# 70076T; RRID: AB_2799771 |
| Rabbit polyclonal CD68 | Affinity | Cat# DF7518; RRID: AB_2841017 |
| Rabbit polyclonal S100A8 | Affinity | Cat# DF6556; RRID: AB_2838518 |
| Rabbit polyclonal RNF128 | Affinity | Cat# DF14658 |
| Rabbit polyclonal RNF128 | Abcam | Cat# ab72533; RRID: AB_1270351 |
| Rabbit polyclonal S100A8 | Abcam | Cat# ab180735 |
| Rabbit polyclonal Tollip | Abcam | Cat# ab187198; RRID: AB_3101868 |
| Mouse polyclonal α-SMA | Abcam | Cat# ab7817; RRID: AB_262054 |
| Rabbit polyclonal CD3E | Abclonal | Cat# A19017; RRID: AB_2862509 |
| Rat Monoclonal Flag-tag | BioLegend | Cat# 637301 |
| Rabbit polyclonal RNF128 | Cell Signaling Technology | Cat# 71590 |
| [HRP-conjugated Goat anti-Rabbit IgG (H+L)](https://abclonal.com.cn/catalog/AS014) | Abclonal | Cat# AS014; |
| HRP-conjugated Goat anti-Mouse IgG (H+L) | Abclonal | Cat# AS003; |
| HRP-conjugated Goat anti-Rat IgG (H+L) | Abclonal | Cat# AS028 |
| CoraLite488-conjugated Goat Anti-Rabbit IgG(H+L) | Proteintech Group | Cat# SA00013-2; RRID: AB_2797132 |
| CoraLite594–conjugated Goat Anti-Mouse IgG(H+L) | Proteintech Group | Cat# SA00013-3; RRID: AB_2797133 |
|  |  |  |

**Table S2 The siRNA sequences**

| **Genes** | **Sequence (5’→3’)** |  |
| --- | --- | --- |
| siBECN1 | GGAGGAAGAGACUAACUCAGG |  |
| siATG12 | GCAGCUUCCUACUUCAAUUGC |  |

**Table S3 The primers for plasmid construction in this study**

| **Genes** | **Primer sequence (5’-3’)** | **Primer type** |
| --- | --- | --- |
| RNF128-Flag | CAAGCTTGCGGCCGCGAATTCATGGGGCCGCCGCCTGGG | Forward |
|  | CAGGGATGCCACCCGGGATCCTTAAGATTTAATTTCTCGAACAGCAGT | Reverse |
| RNF128-Flag_1-276_ | CAAGCTTGCGGCCGCGAATTCATGGGGCCGCCGCCTGGG | Forward |
|  | CAGGGATGCCACCCGGGATCCTCAACTATCTCCATCAGGGCCA | Reverse |
| RNF128-Flag_277-428_ | CAAGCTTGCGGCCGCGAATTCATGTGTGCTGTGTGCATTGAATT | Forward |
|  | CAGGGATGCCACCCGGGATCCTTAAGATTTAATTTCTCGAACAGCAGT | Reverse |
| S100A8-GFP | TCGAGCTCAAGCTTCGAATTCTGTTGACCGAGCTGGAGAAAG | Forward |
|  | ATGGTGGCGACCGGTGGATCCCTCTTTGTGGCTTTCTTCATGGC | Reverse |
| S100A8-GFP_1-46_ | TCGAGCTCAAGCTTCGAATTCTGTTGACCGAGCTGGAGAAAG | Forward |
|  | ATGGTGGCGACCGGTGGATCCGATATACTGAGGACACTCGGTCTCTAGC | Reverse |
| S100A8-GFP_47-93_ | TCGAGCTCAAGCTTCGAATTCTGAGGAAAAAGGGTGCAGACG | Forward |
|  | ATGGTGGCGACCGGTGGATCCCTCTTTGTGGCTTTCTTCATGGC | Reverse |
| pLenti-RNF128-Flag | ATAGAAGACACCGACTCTAGAATGGGGCCGCCGCCTGGG | Forward |
|  | TTTGTAGTCAGCCCGGGATCCAGATTTAATTTCTCGAACAGCAGTCTC | Reverse |
| Flag-p62 | CAAGCTTGCGGCCGCGAATTCATGGCCATGTCCTACGTGAAGG | Forward |
|  | CAGGGATGCCACCCGGGATCCTCACAACGGCGGGGGATG | Reverse |
| Flag-NIX | CAAGCTTGCGGCCGCGAATTCATGTCGTCCCACCTAGTCGAGC | Forward |
|  | CAGGGATGCCACCCGGGATCCTCAGTAGGTGCTGGCAGAGGG | Reverse |
| Flag-NBR1 | CAAGCTTGCGGCCGCGAATTCATGGAACCACAGGTTACTCTAAATGT | Forward |
|  | CAGGGATGCCACCCGGGATCCTTAGAACCAGGAGAATGCTTCACTC | Reverse |
| Flag-OPTN | CAAGCTTGCGGCCGCGAATTCATGTCCCATCAACCTCTCAGCT | Forward |
|  | CAGGGATGCCACCCGGGATCCTAAATGATGCAATCCATCACGTG | Reverse |
| Flag-NDP52 | CAAGCTTGCGGCCGCGAATTCATGGAGGAGACCATCAAAGATCC | Forward |
|  | CAGGGATGCCACCCGGGATCCTCAGAGAGAGTGGCAGAACACG | Reverse |
| Flag-Tollip | CAAGCTTGCGGCCGCGAATTCATGGCGACCACCGTCAGC | Forward |
|  | CAGGGATGCCACCCGGGATCCCTATGGCTCCTCCCCCATCT | Reverse |
| S100A8_K7R_-GFP | ACCGAGCTGGAGAGAGCCTTGAACTCT | Forward |
|  | AGAGTTCAAGGCTCTCTCCAGCTCGGT | Reverse |
| S100A8_K36R_-GFP | GATGACCTGAAGAGATTGCTAGAGACC | Forward |
|  | GGTCTCTAGCAATCTCTTCAGGTCATC | Reverse |

**Table S4 The shRNA and sgRNA sequences**

| **Genes** | **Primer sequence (5’-3’)** | **Primer type** |
| --- | --- | --- |
| shRNF128- #1 | CCGGTCTTAACGTGCAACCATATTTCTCGAGAAATATGGTTGCACGTTAAGATTTTTG | Forward |
|  | AATTCAAAAATCTTAACGTGCAACCATATTTCTCGAGAAATATGGTTGCACGTTAAGA | Reverse |
| shRNF128- #2 | CCGGAGAGACTGCTGTTCGAGAAATCTCGAGATTTCTCGAACAGCAGTCTCTTTTTTG | Forward |
|  | AATTCAAAAAAGAGACTGCTGTTCGAGAAATCTCGAGATTTCTCGAACAGCAGTCTCT | Reverse |
| sgATG5 | CACCGTCCATGAGTTTCCGATTGA | Forward |
|  | AAACTCAATCGGAAACTCATGGAC | Reverse |
| sgATG7 | CACCGAAATAATGGCGGCAGCTACG | Forward |
|  | AAACCGTAGCTGCCGCCATTATTTC | Reverse |

**Table S5 The primers for mice genotyping**

| **Primer** | **Sequence (5’→3’)** | **Primer type** |
| --- | --- | --- |
| P1 | GTTCTCGACACAGAGGGGTC | Forward |
| P2 | AACCCACAAGGTGGAAGGAG | Reverse |
| P3 | TCTCGACACAGAGGGGTCAT | Forward |
| P4 | TGAAATAGCCCACGGTTGCT | Reverse |

**
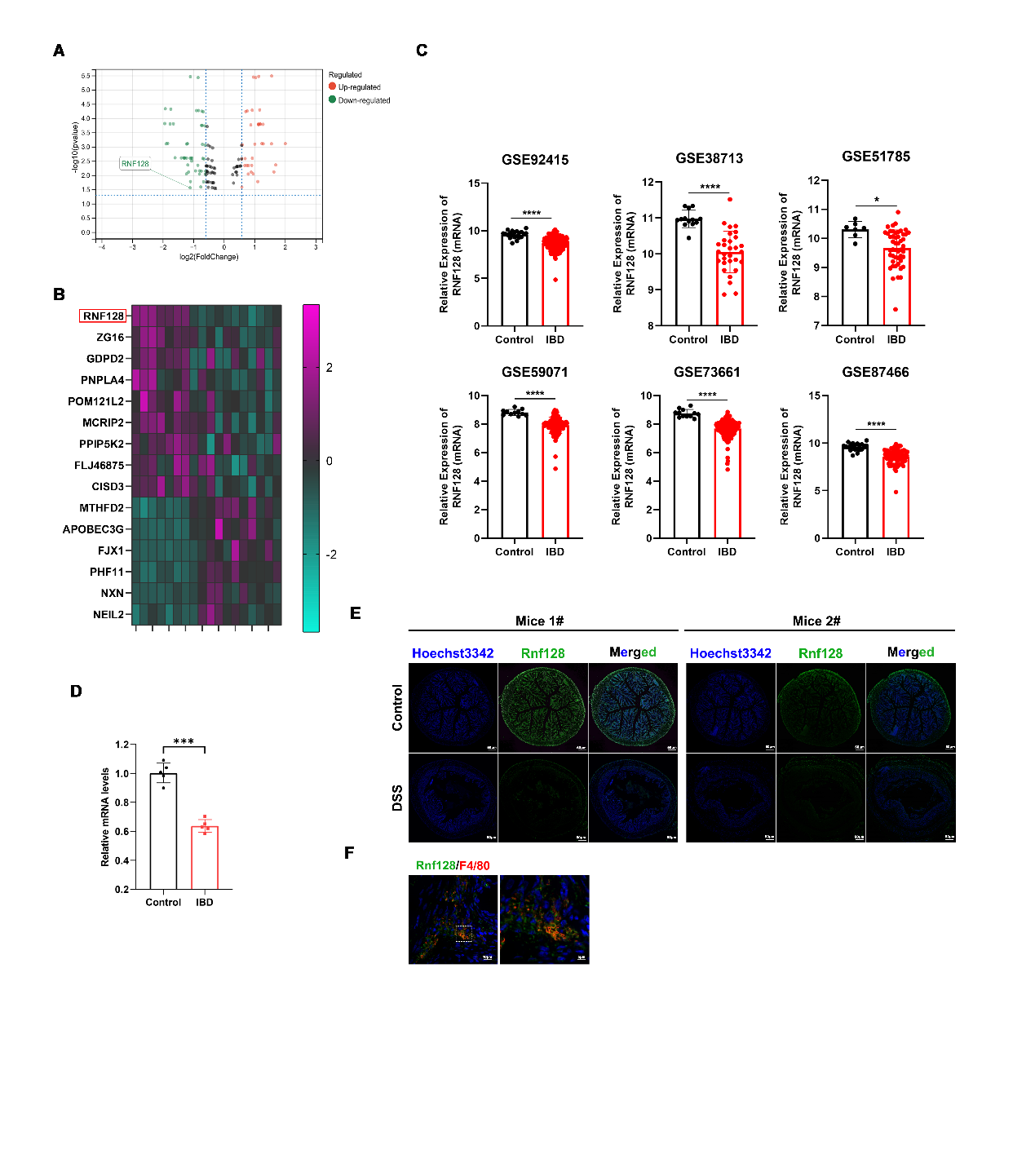
**

**Supplementary Figure 1** (**A**) Volcano map of a portion of the differential genes in IBD tissues and control tissues in GSE4183. Significantly upregulated/downregulated genes (adjusted p < 0.10) were marked in red and blue, respectively. (**B**) The heatmap showed the mRNA change of RNF128 in IBD tissues compared with healthy controls in GSE4183. (**C**) Analysis of *RNF128* mRNA expression in IBD patients compared with healthy controls in GEO database (GSE92415, GSE38713, GSE51785, GSE59071, GSE73661 and GSE87466). * P < 0.05, **** P < 0.0001. (**D**) Analysis of *Rnf128* mRNA expression (normalized to GAPDH RNA levels) in colon tissues from control and DSS-induced colitis mice. *** P < 0.001. (**E**) Representative images of Rnf128 (green) and nuclei (Hoechst 33342, blue) staining in control and DSS-induced colitis mice. Scale bars, 50 µm. (**F**) Representative images of immunofluorescence co-staining for Rnf128 (green) with macrophage markers F4/80 (red) in colitis tissues from DSS-induced colitis mice. DAPI (blue) labels the nuclei. Scale bars, 10 μm.


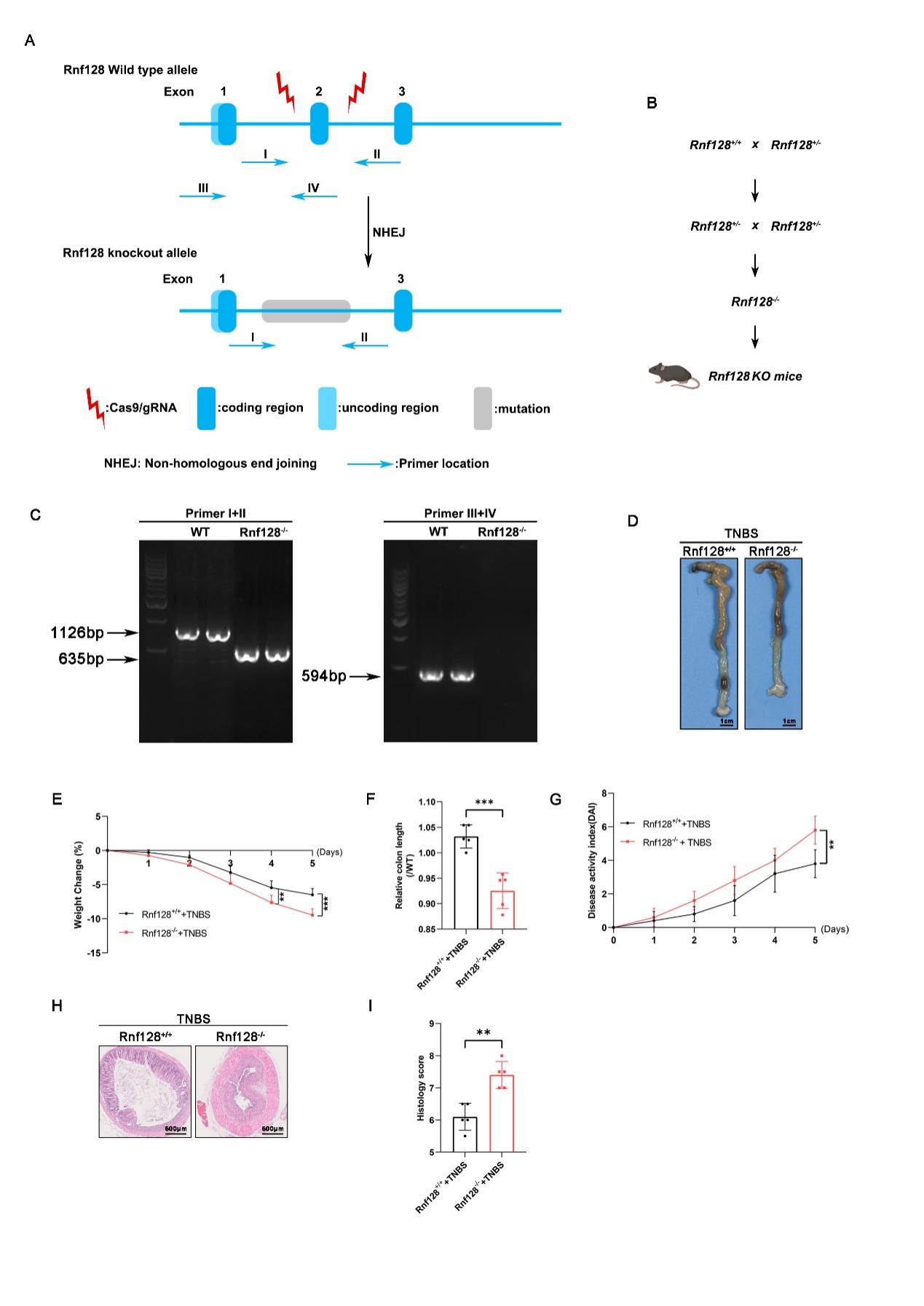


**Supplementary Figure 2.** (**A**) Gene-targeting strategy for generating *Rnf128* knockout mice by CRISPR/Cas9 system. Blue boxes with numbers denoted *Rnf128* exons. Red arrowheads showed Cas9/gRNA sites. (**B**) Crossing strategies for constructing *Rnf128^-/-^* mice. (**C**) The genotyping of *Rnf128^+/+^* and *Rnf128^-/-^* mice was identified by PCR. (D) *Rnf128^+/+^* (n =5) and *Rnf128^-/-^* mice (n = 5) were exposed to 2.5% TNBS for 5 days, representative images of colon were shown. Scale bars, 1 cm. (**E**) Rnf128^+/+^ (n =5) and Rnf128^-/-^ mice (n = 5) were exposed to 2.5% TNBS for 7 days and bodyweight of mice were measured daily. (**F**) The colon length in (D) was measured and analyzed. Statistical data are presented as mean ± SD. *** P < 0.001. (**G**) Disease activity index score in (D) were shown (n = 5). Statistical data are presented as mean ± SD. ** P < 0.01. (**H**) Representative H&E-staining images of colon sections from mice in (D). Scale bar, 500 μm. (**I**) Histopathology score in (H) were measured. Statistical data are presented as mean ± SD. ** P < 0.01.


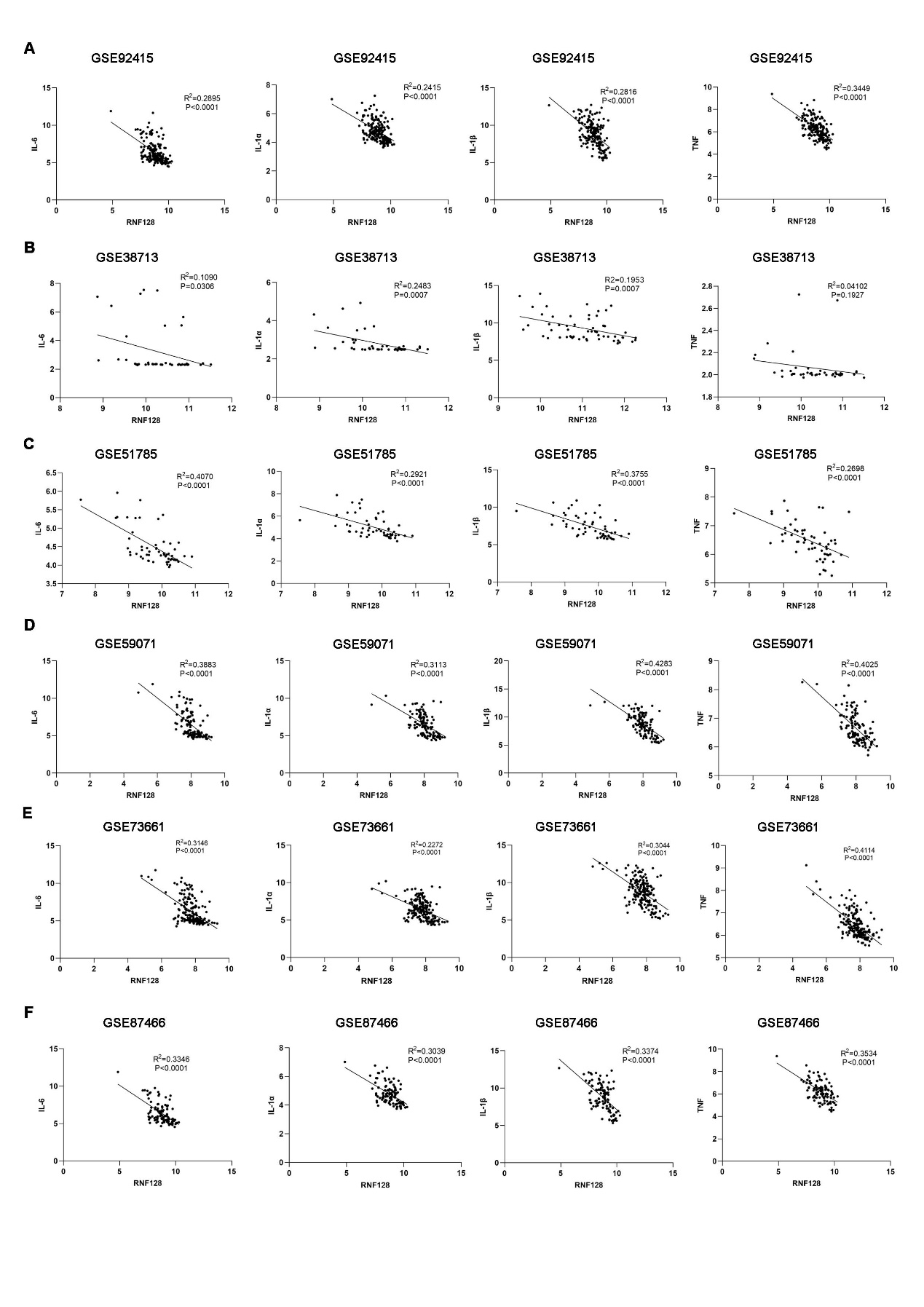


**Supplementary Figure 3.** (**A-F**) The correlation between the mRNA expression of *RNF128* and IL-6, IL-1α, IL-1β, TNF-α in GSE92415 (A), GSE38713 (B), GSE51785 (C), GSE59071 (D), GSE73661 (E) and GSE87466 (F).


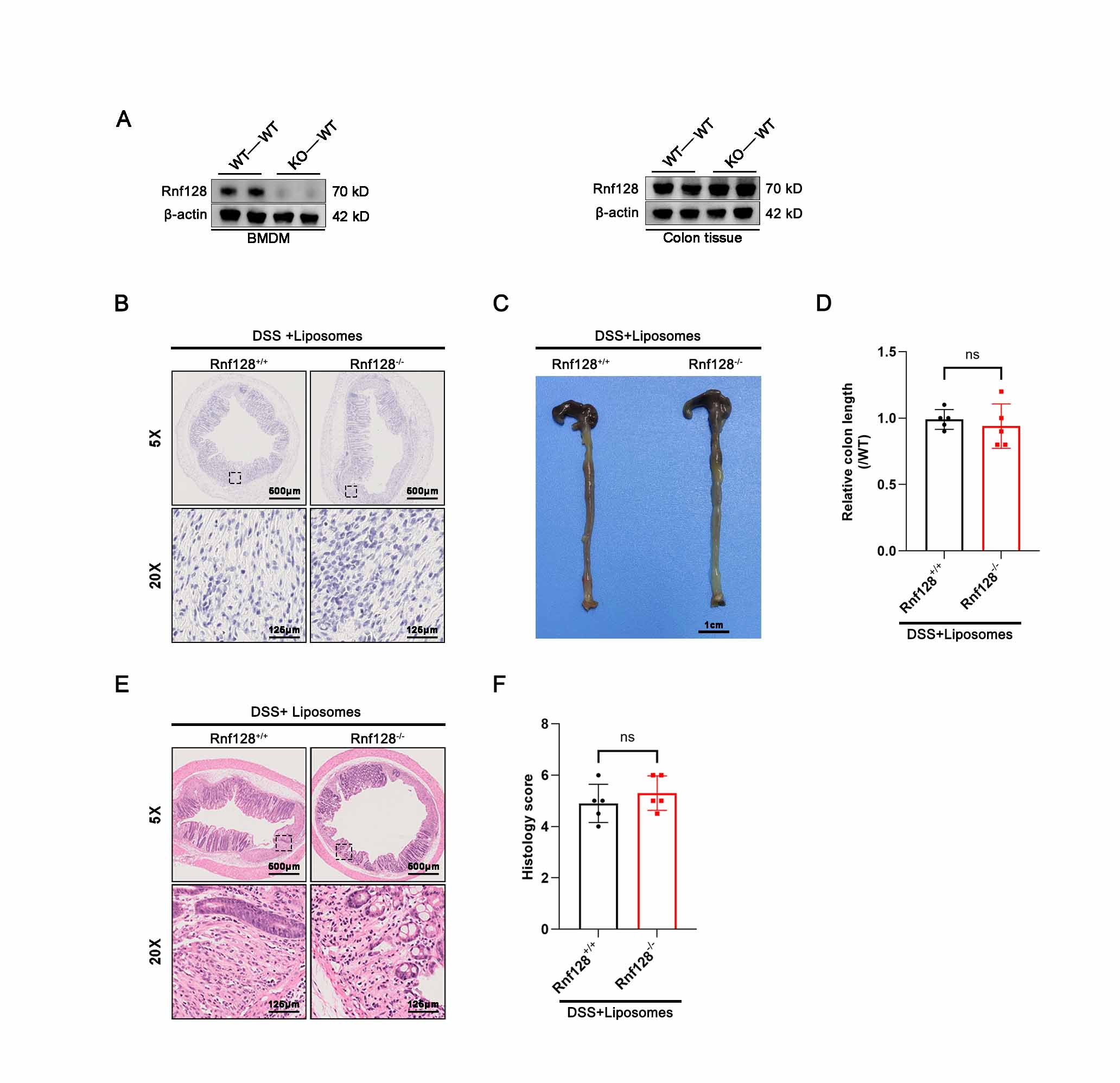


**Supplementary Figure 4.** (**A**) Bone marrow derived macrophages (BMDMs) and colon tissues were obtained from chimeric mice 7 weeks after the transplantation of bone marrow cells. (**B**) Representative F4/80 staining images of colon sections from mice. Scale bar, 500 μm. (**C**) *Rnf128^+/+^* (n =5) and *Rnf128^-/-^* mice (n = 5) were treated with Liposome for 3 times before exposing to 2.5% DSS for 7 days. Representative images of colon were shown Scale bars, 1 cm. (**D**) The colon length in (C) was measured and analyzed. Statistical data are presented as mean ± SD. ns, nonsense. (**E**) Representative H&E-staining images of colon sections from mice in (C). Scale bar, 500 μm. (**F**) Histopathology score in (E) were measured. Statistical data are presented as mean ± SD. ns, nonsense.


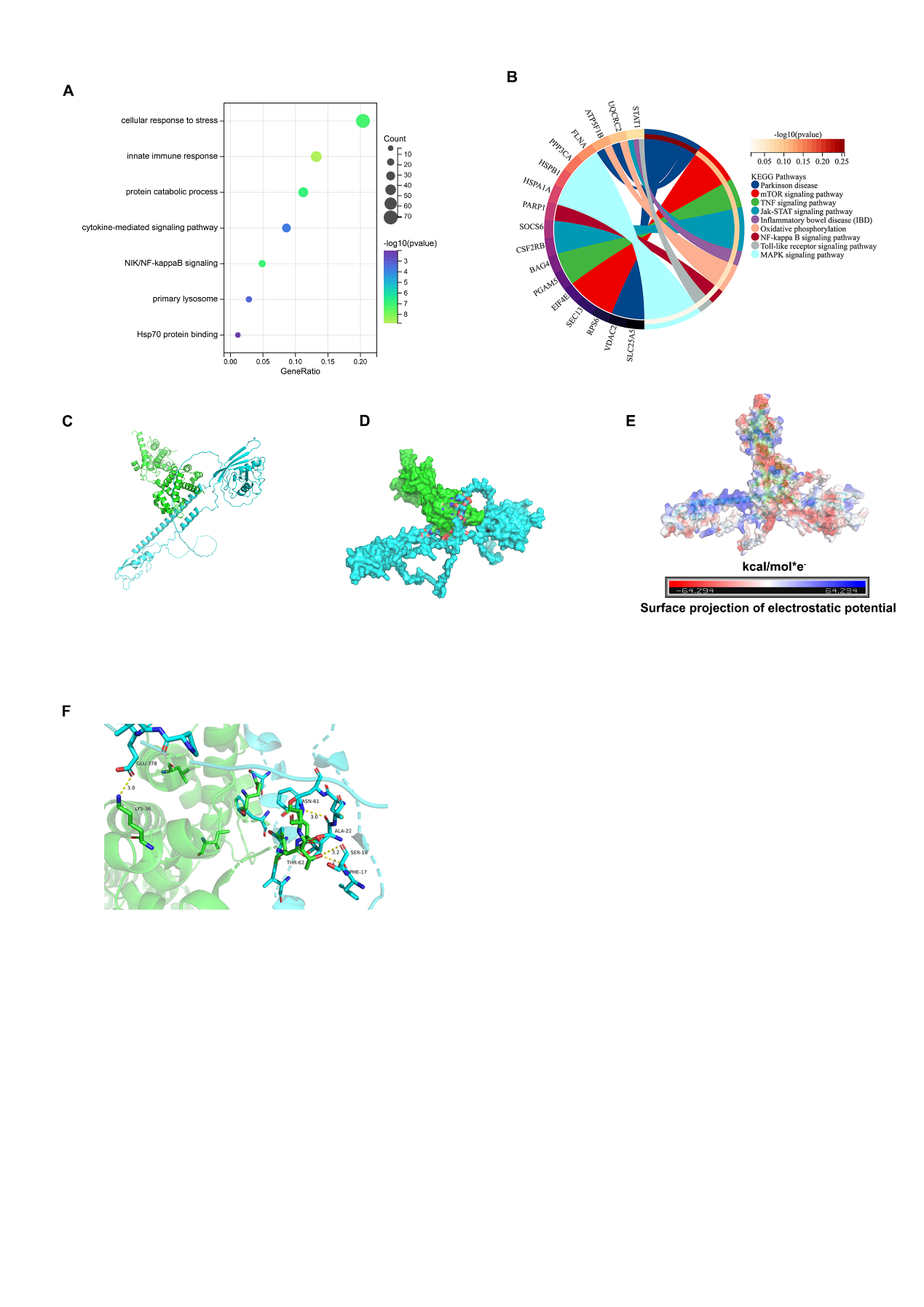


**Supplementary Figure 5.** (**A**) Enriched GO terms of proteins interacting with RNF128. (**B**) KEGG pathway analysis of proteins interacting with RNF128. (**C**) AlphaFold model of the RNF128 (cyan) bound to the S100A8 (green). (**D**) The surface image of binding conformation between RNF128 (cyan) and S100A8 (green). The blue and red areas are the binding region. (**E**) Electrostatic potential mapped on to the surface of the RNF128 bound to the S100A8. (**F**) PyMol software for the visualization of amino acid residues in the interaction of RNF128 (cyan) with S100A8 (green).


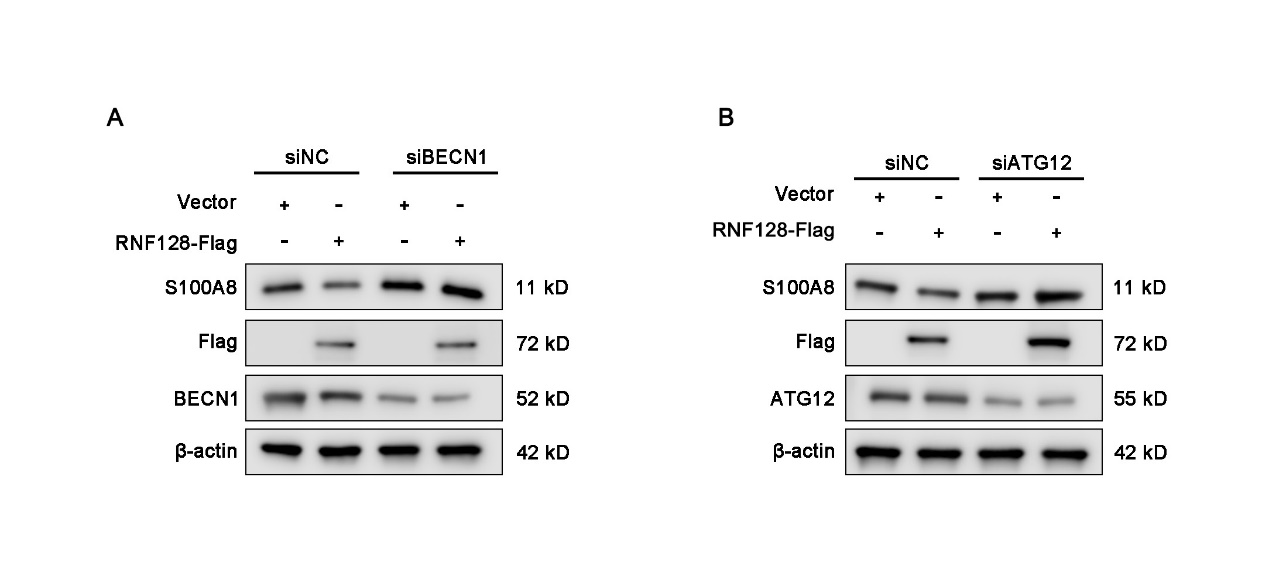


**Supplementary Figure 6.** (**A**) Control siRNA or BECN1 siRNA were transfected into RNF128 overexpressing THP-1 cells for 48h. The expression of S100A8 was measured by western blot. (**B**) Control siRNA or ATG12 siRNA were transfected into RNF128 overexpressing THP-1 cells for 48h. The expression of S100A8 was measured by western blot.


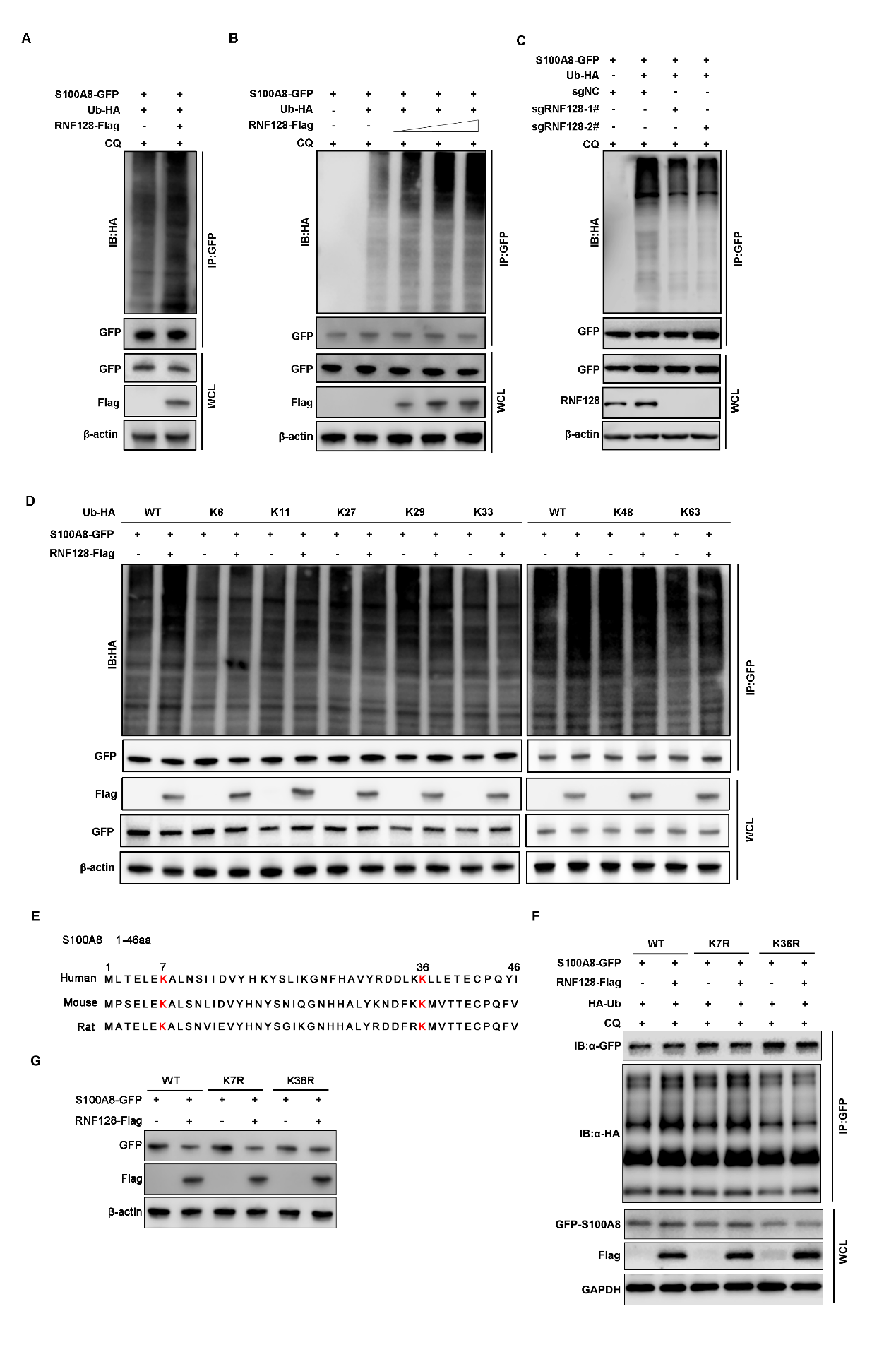


**Supplementary Figure 7** (**A**) 293T cells were co-transfected with HA-ubiquitin (Ub), S100A8-GFP and RNF128-Flag plasmids for 48h, and then treated with CQ (50 nM) for another 4 h. Total lysates were immunoprecipitated with anti-GFP antibody. The immunoprecipitation complex was analyzed by GFP and HA antibody. (**B**) 293T cells were co-transfected with HA-ubiquitin (Ub), S100A8-GFP and different concentration of RNF128-Flag plasmids, and then treated with CQ (50 nM) for another 4 h. The total lysates were immunoprecipitated with anti-GFP antibody. The immunoprecipitation complex was analyzed by GFP and HA antibody. (**C**) Control or RNF128 stably knockout THP-1 cells were transfected with HA ubiquitin (Ub) and S100A8-GFP plasmids for 48h, and then treated with CQ (50 nM) for another 4 h. Total lysates were immunoprecipitated with anti-GFP antibody. The immunoprecipitation complex was analyzed by GFP and HA antibody. (**D**) 293T cells were transfected with HA-ubiquitin mutants (K6, K11, K27, K33, K48, or K63), S100A8-GFP, and RNF128-Flag plasmids for 48h, and then treated with CQ (50 nM) for another 4 h. Total lysates were immunoprecipitated with anti-GFP antibody. The immunoprecipitation complex was analyzed by GFP and HA antibody. (**E**) Alignment of S100A8_1-46_ amino acid sequences in Human, mouse and rat. Highlighted amino acids indicate conserved lysine (K) of S100A8_1-46_. (**F**) 293T cells were transfected with HA- ubiquitin (Ub), S100A8-GFP mutants (K7R, K36R) and RNF128-Flag plasmids for 48h, and then treated with CQ (50 nM) for another 4 h. Total lysates were immunoprecipitated with anti-GFP antibody. The immunoprecipitation complex was analyzed by GFP and HA antibody. (**G**) THP-1 cells were transfected with RNF128-Flag and S100A8-GFP WT or S100A8 mutants (K7R, K36R). The expression of S100A8-GFP were analyzed by western blot.
