# Supplementary material for: RNF128 deficiency in macrophages promotes colonic inflammation by suppressing the autophagic degradation of S100A8
Source: Cell Death Dis. 2025 Jan 15;16(1):20. doi: 10.1038/s41419-025-07338-0 (PMC11733159; doi:10.1038/s41419-025-07338-0)

**Original image for Figure 1**


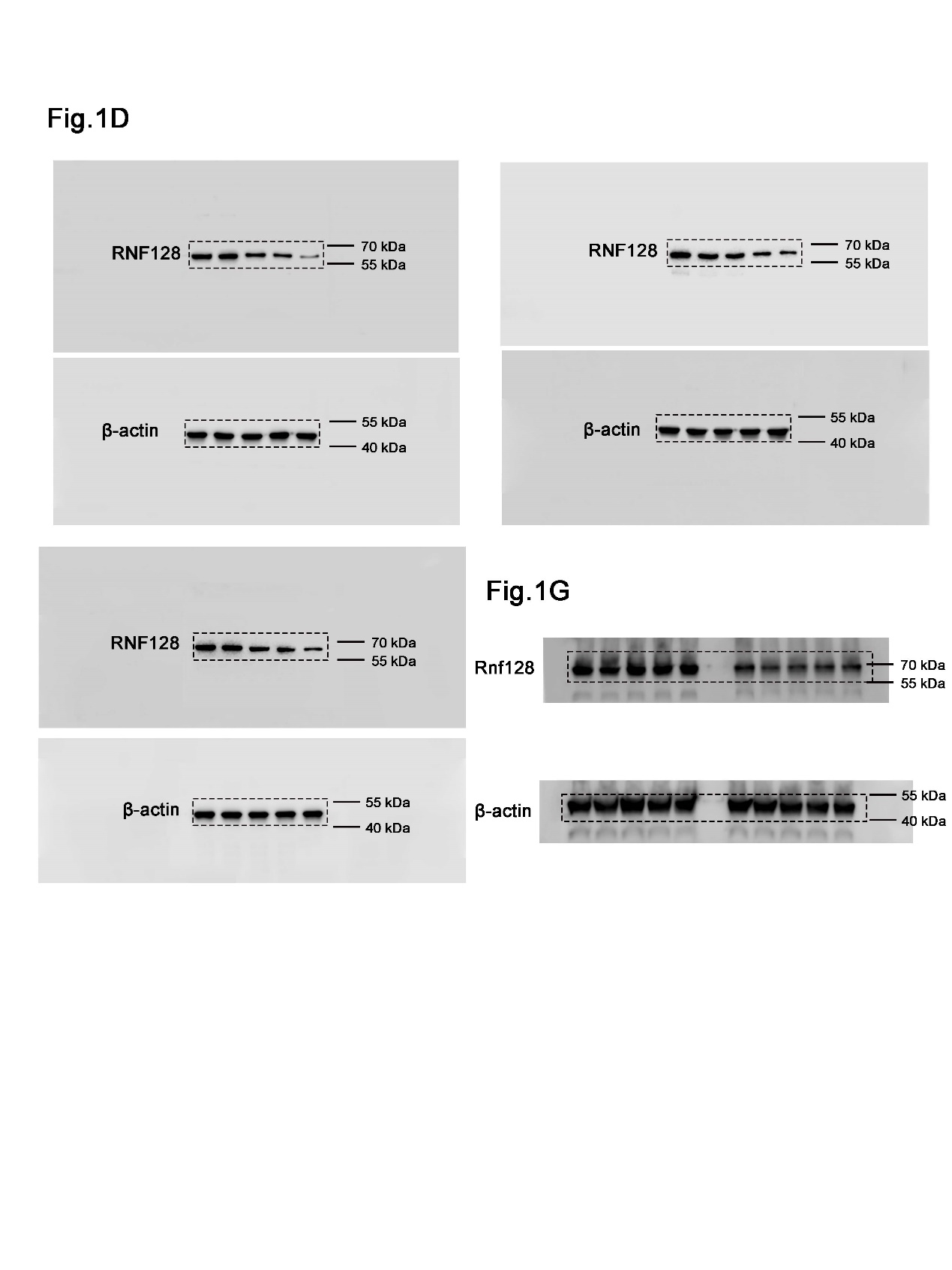


**Original image for Figure 5**


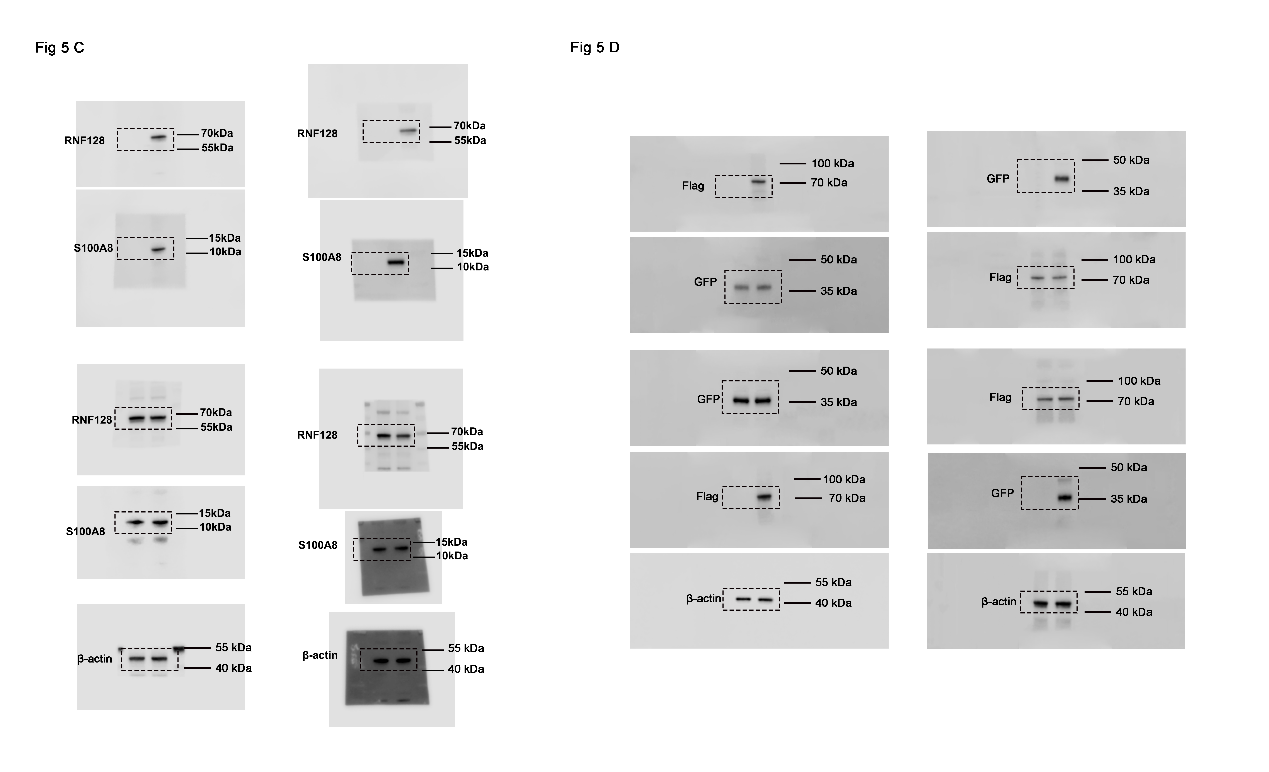


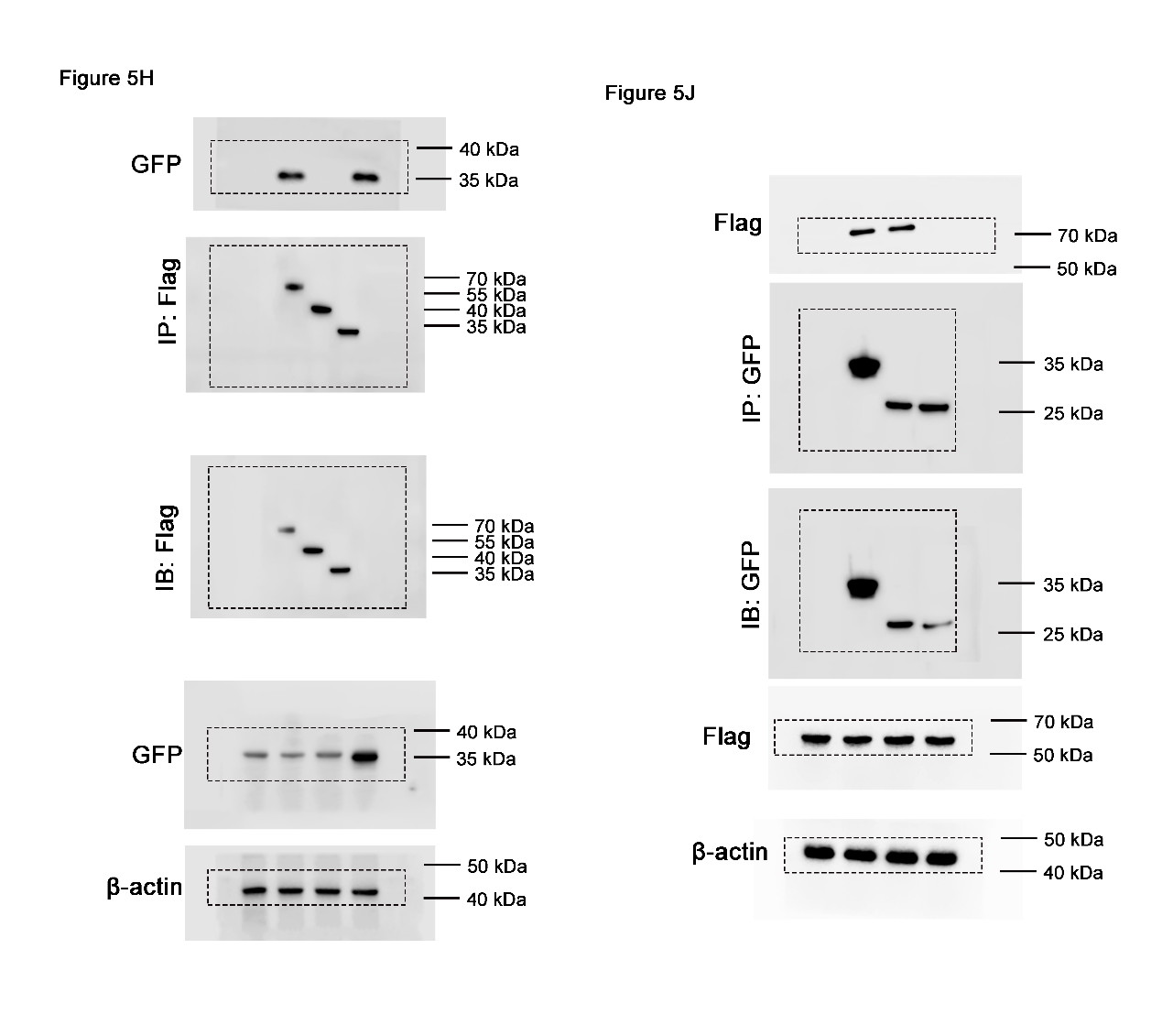


**Original image for Figure 6**


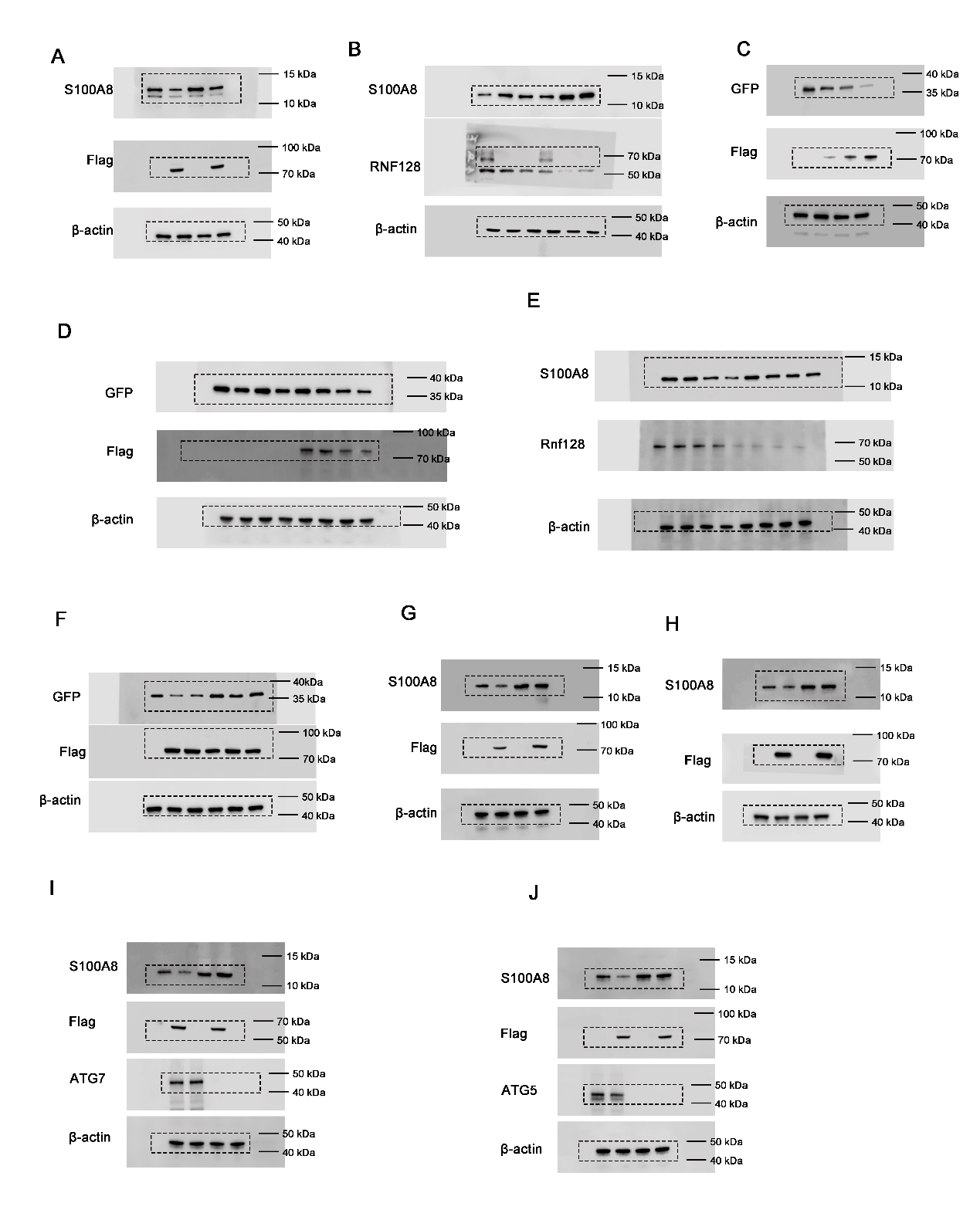


**Original image for Figure 7**


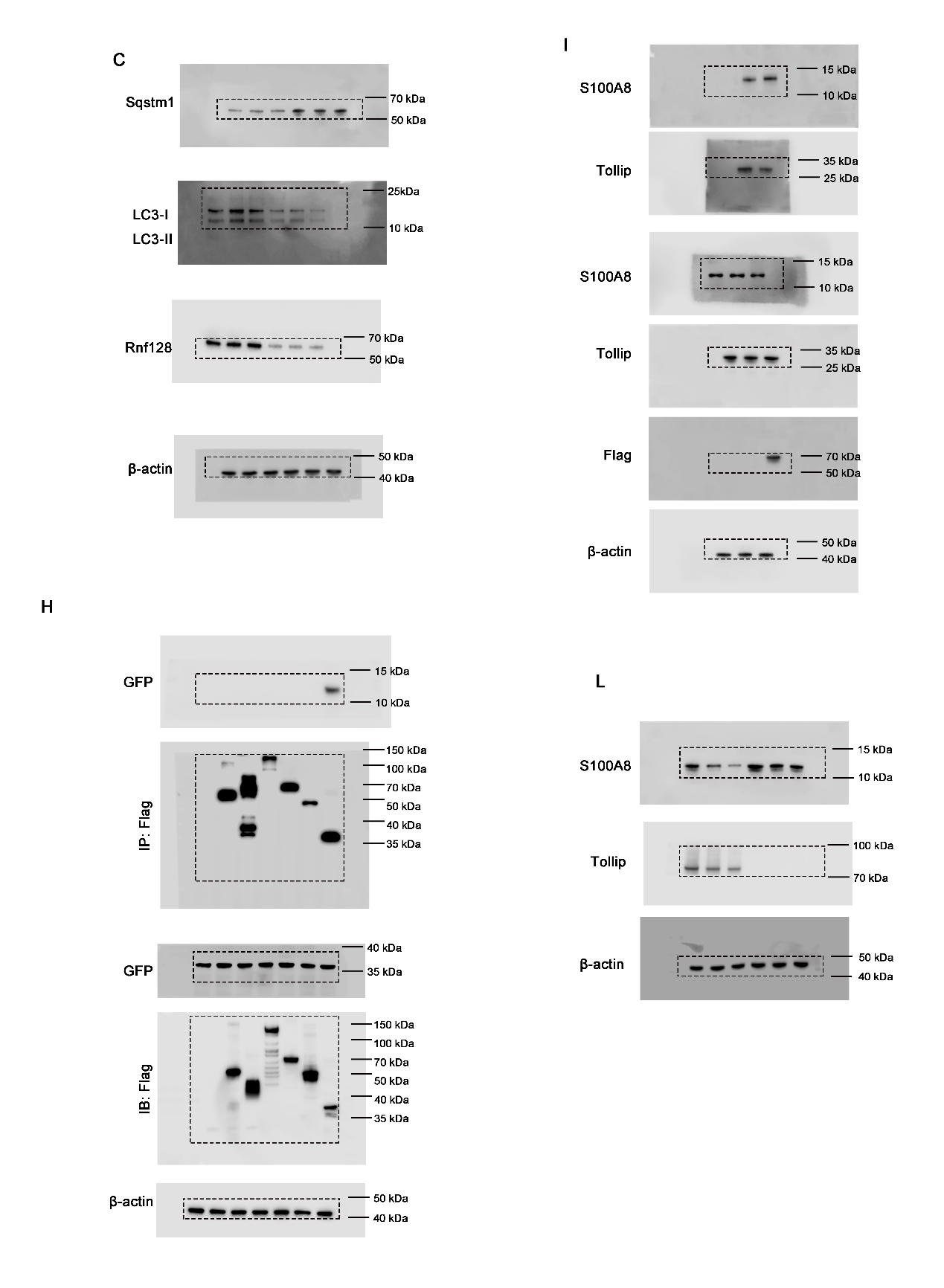


**Original image for Figure S4**


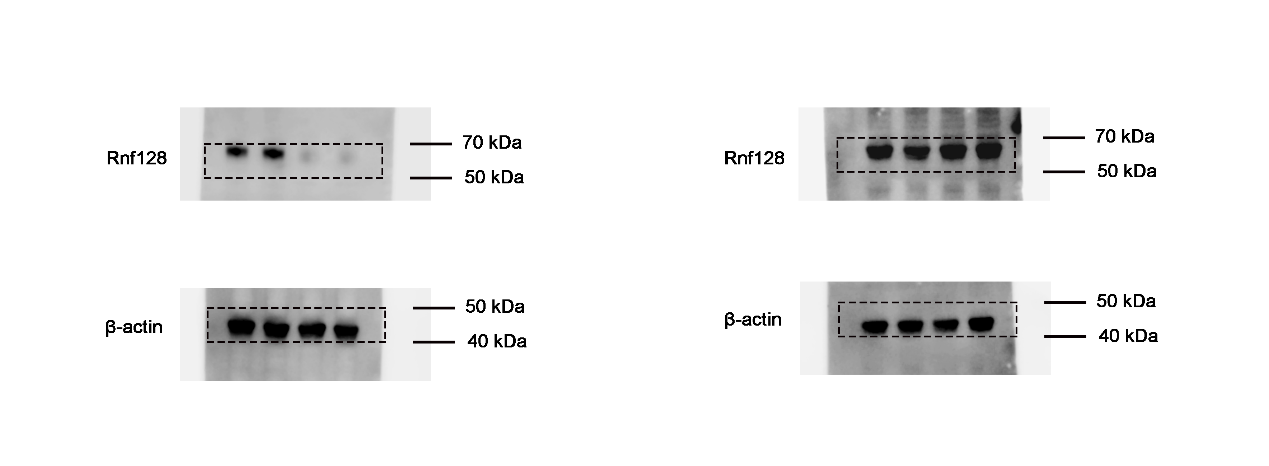


**Original image for Figure S6**


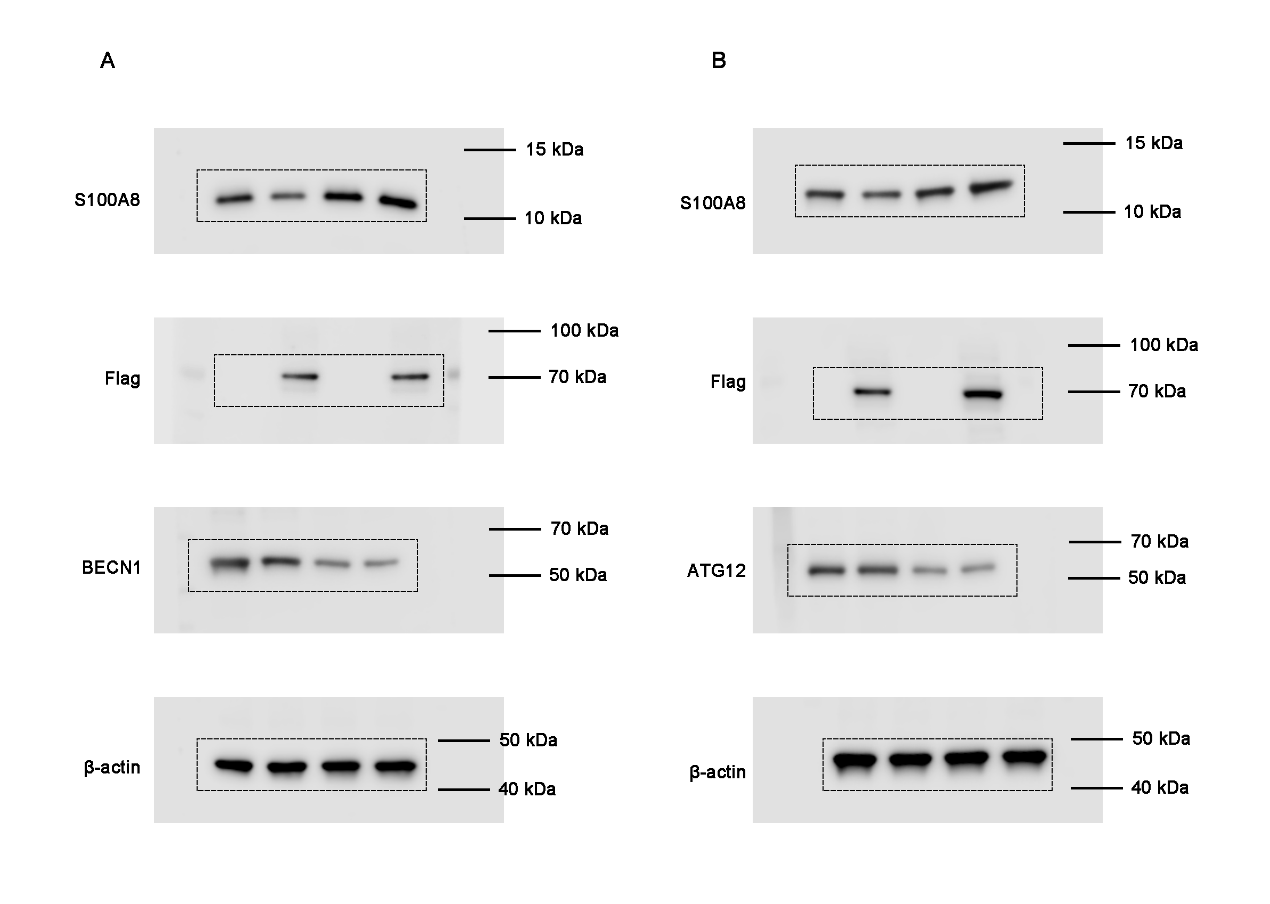


**Original image for Figure S7**


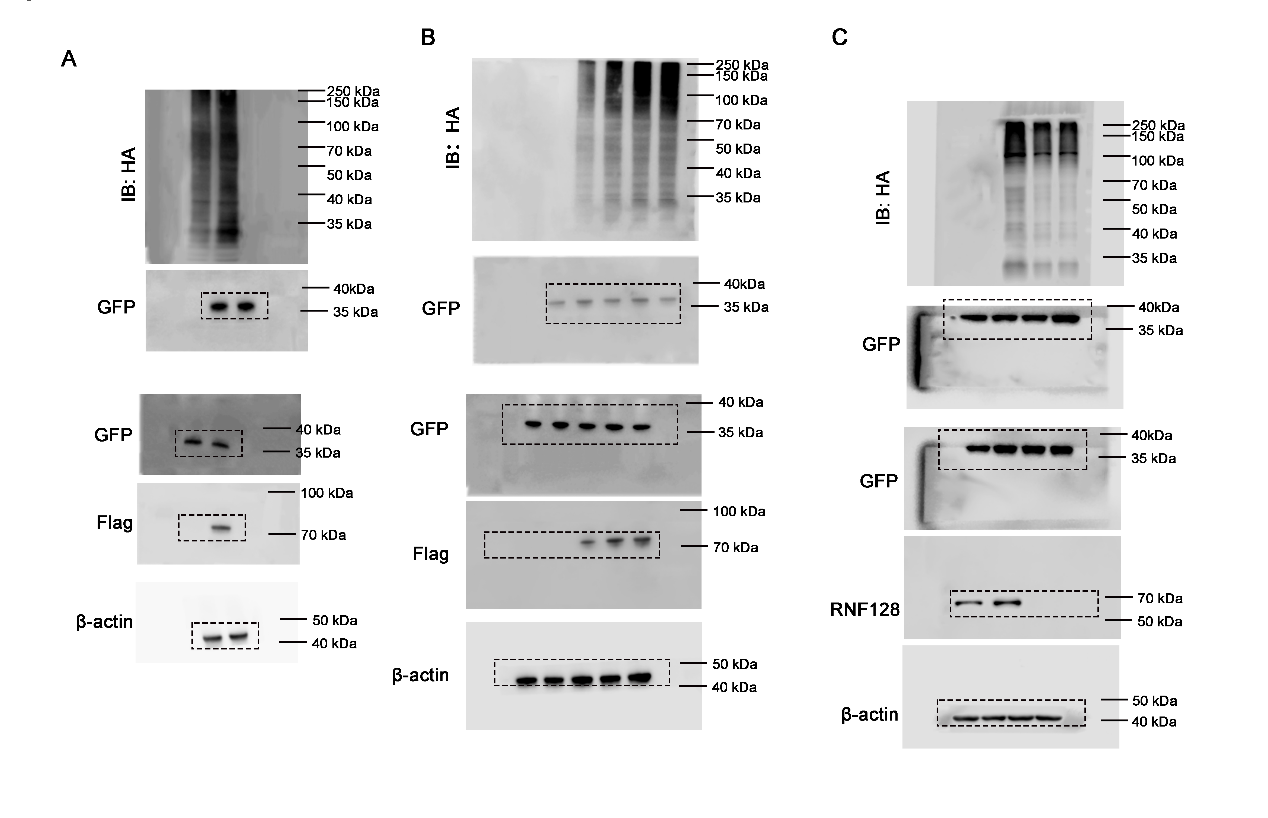


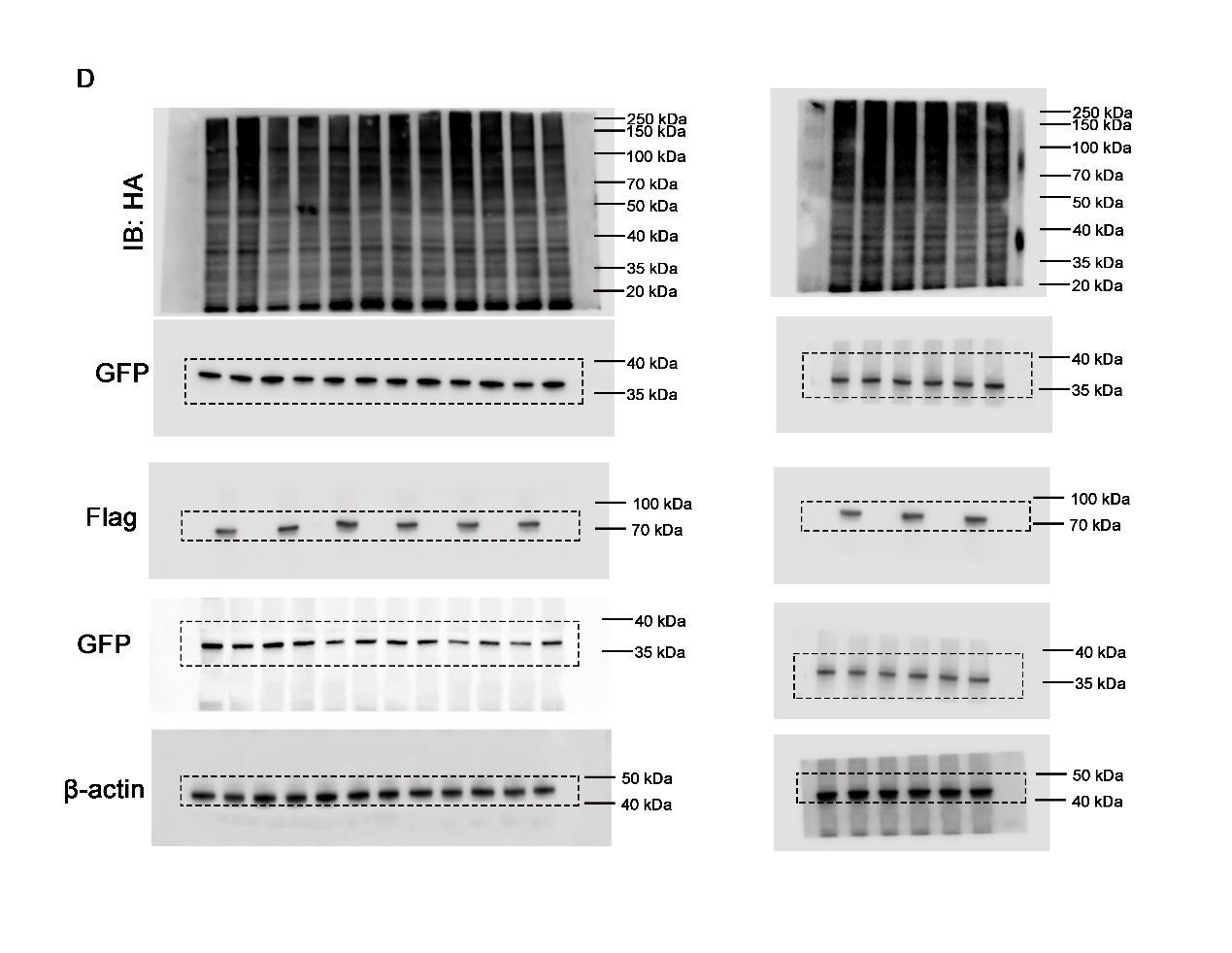


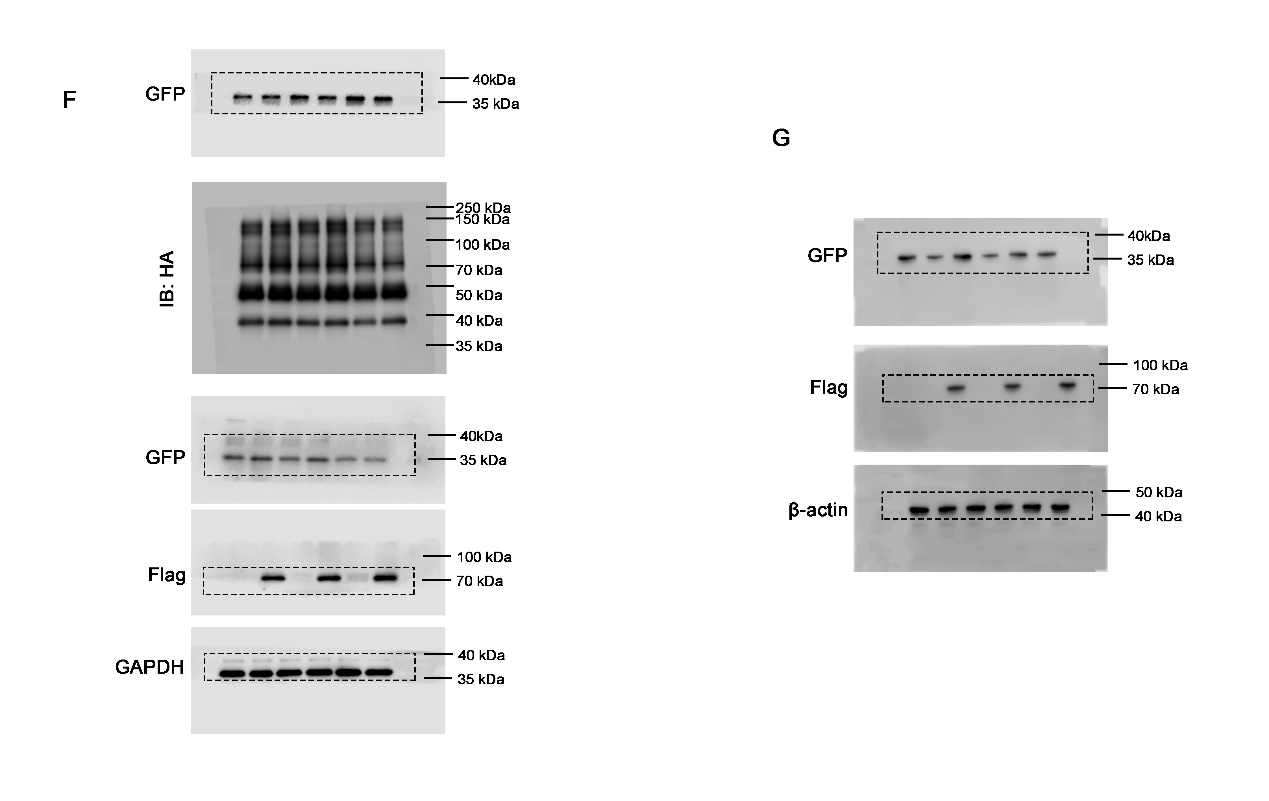

Supplement: Supplementary file 2 — Original image for western blot [file 41419_2025_7338_MOESM2_ESM.docx]
